# Sequential targeting of YAP1 and p21 enhances the elimination of senescent cells induced by the BET inhibitor JQ1

**DOI:** 10.1038/s41419-021-03416-1

**Published:** 2021-01-25

**Authors:** Huan-Tian Zhang, Tao Gui, Ri-Xu Liu, Kui-Leung Tong, Chong-Jie Wu, Zhenyan Li, Xun Huang, Qiu-Tong Xu, Jie Yang, Wang Tang, Yuan Sang, Wanting Liu, Ning Liu, Ryan D. Ross, Qing-Yu He, Zhen-Gang Zha

**Affiliations:** 1 Institute of Orthopedic Diseases, Jinan University, Guangzhou, China; 2 Center for Joint Surgery and Sports Medicine, the First Affiliated Hospital, Jinan University, Guangzhou, China; 3 Key Laboratory of Functional Protein Research of Guangdong Higher Education Institutes, College of Life Science and Technology, Jinan University, Guangzhou, China; 4 Shandong Provincial Key Laboratory of Detection Technology for Tumor Markers, College of Chemistry and Chemical Engineering, Linyi University, Linyi, China; 5 Department of Joint Replacement and Trauma Surgery, the Third Affiliated Hospital, Sun Yat-sen University, Guangzhou, China; 6 Department of Cell and Molecular Medicine, Rush University Medical Center, Chicago, IL USA

**Keywords:** Sarcoma, Senescence

## Abstract

Chondrosarcoma (CHS) is the second most common bone malignancy with limited therapeutic approaches. Our previous study has found that Yes associated protein 1 (YAP1) is downregulated in CHS cells treated with bromodomain and extraterminal domain (BET) inhibitor JQ1. However, the precise role of YAP1 in CHS is largely unknown. Herein, we found that YAP1 expression was upregulated in CHS tissues, and positively correlated with its grading score. Loss of YAP1 inhibited CHS proliferation and induced cellular senescence, while expression of YAP1 mutants revealed YAP1/TEA domain family member (TEAD)-dependent negative regulation of p21 and subsequent cellular senescence. These results were validated by in vivo experiments using stable shYAP1 cell lines. Mechanistically, negative regulation of p21 by YAP1 occurred post-transcriptionally via Dicer-regulated miRNA networks, specifically, the miR-17 family. Furthermore, we demonstrated that sequential targeting of YAP1 and p21 enhanced the elimination of JQ1-induced senescent cells in a Bcl-2-like 1 (Bcl-XL)/Caspase-3 dependent manner. Altogether, we unveil a novel role of YAP1 signaling in mediating CHS cell senescence and propose a one-two punch approach that sequentially targets the YAP1/p21 axis to eliminate senescent cells.

## Introduction

Chondrosarcoma (CHS) is the second most common primary bone malignancy and is characterized by the synthesis of hyaline cartilage. Poor vascularization, slow division rate, and substantial hyaline cartilage production confer resistant to conventional chemotherapy and radiotherapy^1^. Over the past 3 decades, complete surgical resection with wide margins remains the major treatment option. Therefore, there is an urgent need to understand the molecular mechanisms that lead to chemo- and radioresistance to develop efficient therapeutic strategies. Several ongoing phases I/II clinical trials have been conducted for CHS, including agents targeting isocitrate dehydrogenase (IDH) mutations, the PI3K-Akt-mTOR, hedgehog, and angiogenesis signaling^2^. In addition, previous studies have highlighted the importance of anti-apoptotic proteins, such as B-cell leukemia/lymphoma 2 (Bcl-2) and Bcl-2-like 1 (Bcl-XL), in mediating cell survival upon chemotherapy and radiotherapy^3,4^. Recently, aberrant expression of Yes-associated protein 1 (YAP1) has been observed in a panel of sarcomas including CHS^5–7^, yet the precise role of YAP1 in CHS remains to be explored.

YAP1 is a mechanosensitive transcriptional regulator that is downstream of the Hippo pathway, which, controls development and tumorigenesis through regulating cell proliferation, survival, differentiation, and cytoskeletal remodeling^8^. The shuttling of YAP1 between the cytoplasm and nucleus is achieved in a phosphorylation-dependent manner (S61, S109, S127, S164, and S381)^9^. Nuclear YAP1 has been shown to interact with different transcription factors, particularly TEA domain family members (TEAD), thereby activating many genes related to cell proliferation and anti-apoptosis effects^10^. Accumulated evidences suggest that nuclear YAP1 plays a crucial role in inhibiting cell senescence in fibroblasts or cancer cells^11,12^, yet others have demonstrated that hyperactivation of YAP1 can induce cell senescence^13^. The diverse roles of YAP1 in cancers can be attributed to a wide range of regulatory miRNA networks^14^; however, whether YAP1 plays a role in regulating cellular senescence via miRNAs is largely unknown.

Cellular senescence is defined as irreversible growth arrest and the production of a bioactive secretome, which has been recognized as a tumor-suppressive mechanism^15^. Based on this, the induction of senescence in cancer cells is an emerging therapeutic option. However, recent studies have suggested that the accumulation of senescent cells in tissues and organs exerts detrimental effects, e.g., by driving secondary tumors or triggering cancer relapse^16,17^. Therefore, eliminating the senescent cells in a precise time window enables the maximization of therapeutic efficacy. Two canonical pathways, the p53/p21 or the pRb/p16 axes, have been implicated in cellular senescence induced by various insults, including telomere shortening, oncogenic signaling, and therapeutic or stress-induced DNA^18,19^. Under therapeutic pressure, p21 expression is greatly induced, which controls cancer cell senescence, however, the substantial expression of p21 can also be detrimental to cancer recurrence^20^. For instance, persistent expression of p21 causes genomic instability in a p53-independent manner^21^. Moreover, p21 expression favors the survival of senescent cells via c-Jun N-terminal kinase (JNK) and caspase signaling^22^. These studies indicate that restricting p21 expression might provide an option for precisely eliminating senescent cells.

In the present study, we systematically evaluated the oncogenic role of YAP1 in CHS both in vitro and in vivo and demonstrated that YAP1 was essential for the modulation of CHS cell senescence by targeting the miR-93/p21 axis. Furthermore, we uncovered that the YAP1/p21 axis was implicated in chemotherapy-induced senescence and proposed sequentially targeting YAP1 and p21 as a “one-two punch” approach to maximize therapeutic efficacy by enhancing the elimination of senescent cells.

## Results

### Nuclear localization of YAP1 confers oncogenic phenotypes in CHS cells

**Fig. 1:**
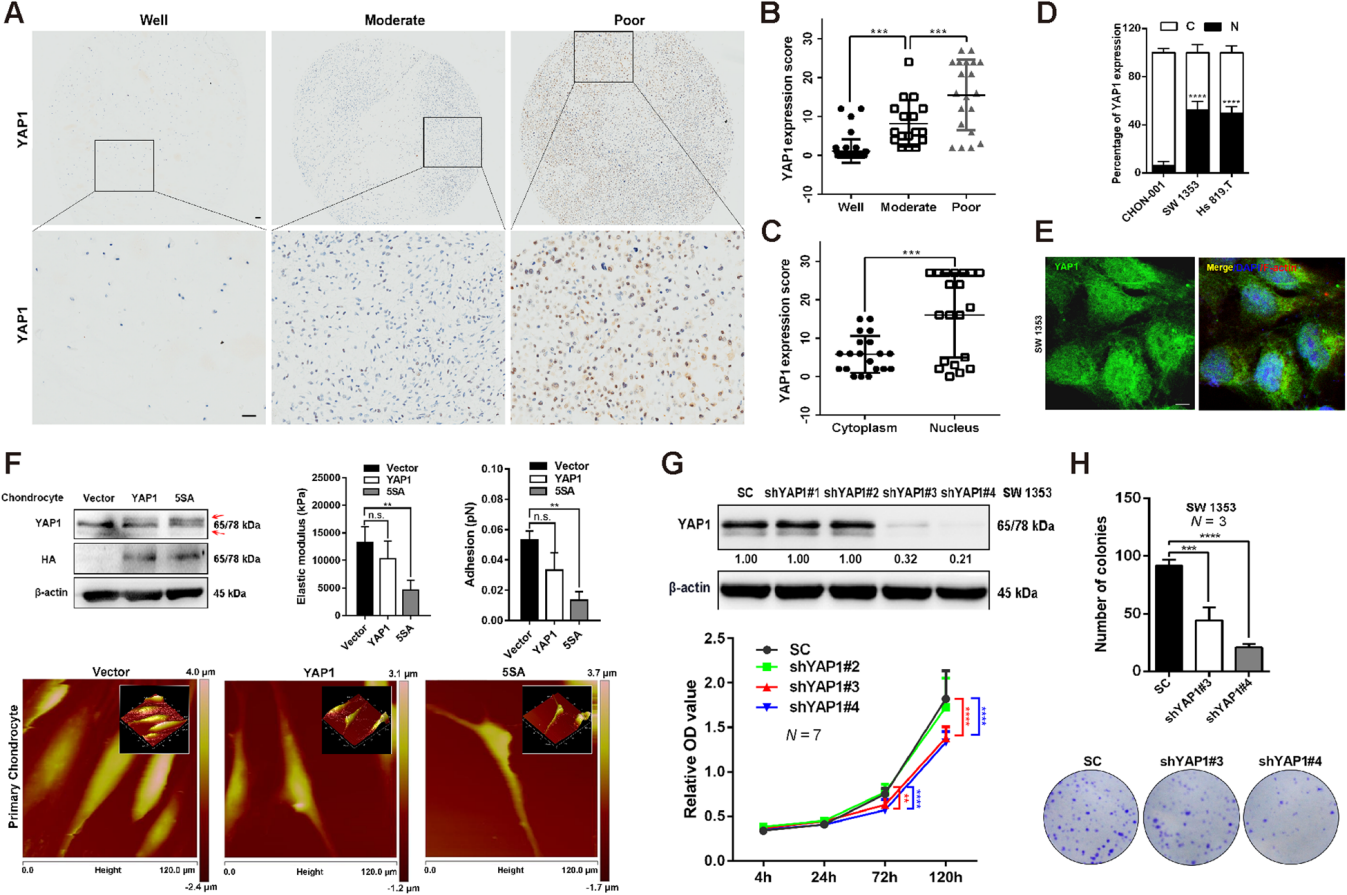
Nuclear YAP1 is required for the growth of CHS cells. **A** IHC analysis of YAP1 expression in tissue arrays of CHS of different grades. Representative images are shown. Scale bar: 50 μm. Well (well-differentiated, *N* = 43), moderate (moderately differentiated, = 17), poor (poorly differentiated, *N* = 20). **B** Quantification of YAP1 expression scores in (**A**). **C** The expression scores of YAP1 in the cytoplasm and nucleus in poorly differentiated CHS (Poor) were quantified. **D** The percentage of YAP1 expression in the cytoplasm and nucleus in the chondrocytic cell line (CHON-001) and CHS cell lines (SW 1353 and Hs 819.T). C: cytoplasm, N: nucleus. **E** Representative IF image of YAP1 localization (green) in SW 1353 cells. DAPI (blue) indicates the nucleus, while F-actin (red) defines the cytoskeleton. Scale bar: 10 μm. **F** IB analysis of YAP1 and HA-tagged expression in primary chondrocytes infected with lentivirus-delivered HA-vector (Vector), HA-YAP1 (YAP1), or HA-YAP1-5SA (5SA). Red arrows indicated both the endogenous and exogenous YAP1 expression. The elastic modulus and adhesion properties of the indicated cells were analyzed by AFM for at least three individual cells; representative images are shown below. **G** SW 1353 cells stably expressing control shRNA or YAP1-specific shRNAs (shYAP#1, #2, #3, #4) were established, and the knockdown efficiency was confirmed using an antibody against YAP1 for IB (above). The cell viabilities of each construct at varying time points were determined by a CCK-8 assay (below). **H** Colony formation ability (number of colonies) of the indicated stable cells is shown (above). Representative images are shown below. Data are presented as the mean ± SD of at least three independent experiments in panel (**B**–**D**, **F**–**H**). One-way ANOVA followed by Dunnett’s test was applied for (**B**, **F**, **H**). Student’s *t*-test for (**C**, **D**). Two-way ANOVA followed by Tukey’s test for (**G**). n.s. nonsignificant, *P < 0.05, **P < 0.01, ***P < 0.001, ****P < 0.0001.

YAP1, a critical downstream effector of the Hippo pathway, has been reported to be involved in the development and progression of several human cancers including sarcoma^23^, yet its roles in CHS are largely unknown. Regardless of anatomical location, all CHS are classified by histology into three grades ranging from well-differentiated grade I to poorly differentiated grade III, of which 70% of cases develop metastasis^24^. This study firstly evaluated the expression of YAP1 in a series of CHS tissues. Notably, the expression of YAP1 was significantly increased with tumor grade from well to poor (Fig. 1A, B). We also observed a predominant localization of YAP1 in the nucleus in CHS tissues (Fig. 1C), which was in agreement with previous findings that YAP1 localizes in the nucleus, thereby initiating oncogenic signalings^25,26^. Interestingly, the percentage of nuclear YAP1 was higher in the CHS cells (SW 1353 and Hs 819.T) than that in the chondrocytic cell line, CHON-001 (Fig. 1D, E and Fig. S1a). Overexpression of constitutively activated YAP1 (5SA, located in the nucleus) using lentivirus led to a slender cell morphology change in the CHON-001 cells (Fig. S1b). Moreover, 5SA overexpression resulted in a remarkable suppression of F-actin networks, which are critical factors in the determination of cell stiffness, in human primary chondrocytes in a cell density-independent manner (Fig. S1c). In addition, single-cell AFM analysis demonstrated that elastic modulus and cell adhesion, which are hallmarks of oncogenic transformation^27^, were significantly attenuated by enforced YAP1 5SA expression (Fig. 1F and Fig. S1d).

In contrast, transient knockdown of YAP1 by the siRNAs (siY#1, siY#2, and siY#4) significantly abolished the growth of SW 1353 cells (Fig. S1e). Consistently, knockdown of YAP1 using shRNAs (i.e., shYAP1#3 and #4) led to significant inhibition of the SW 1353 cell growth and colony formation, despite a compensatory increase in expression of its heterodimer, TAZ (Fig. 1G, H and Fig. S1f). Interestingly, YAP1/TAZ double knockdown did not show the synergistic effect on suppressing cell growth, indicating that YAP1 was the major effector in the SW 1353 cells (Fig. S1g). Similarly, inhibition of colony formation by YAP1 knockdown was observed in another CHS cell line, Hs 819.T (Fig. S1h). Altogether, these data reveal that nuclear YAP1 is required for the acquisition of oncogenic phenotypes and the growth of CHS cells.

### YAP1 and TEAD control cell cycle exit and induce cellular senescence

**Fig. 2:**
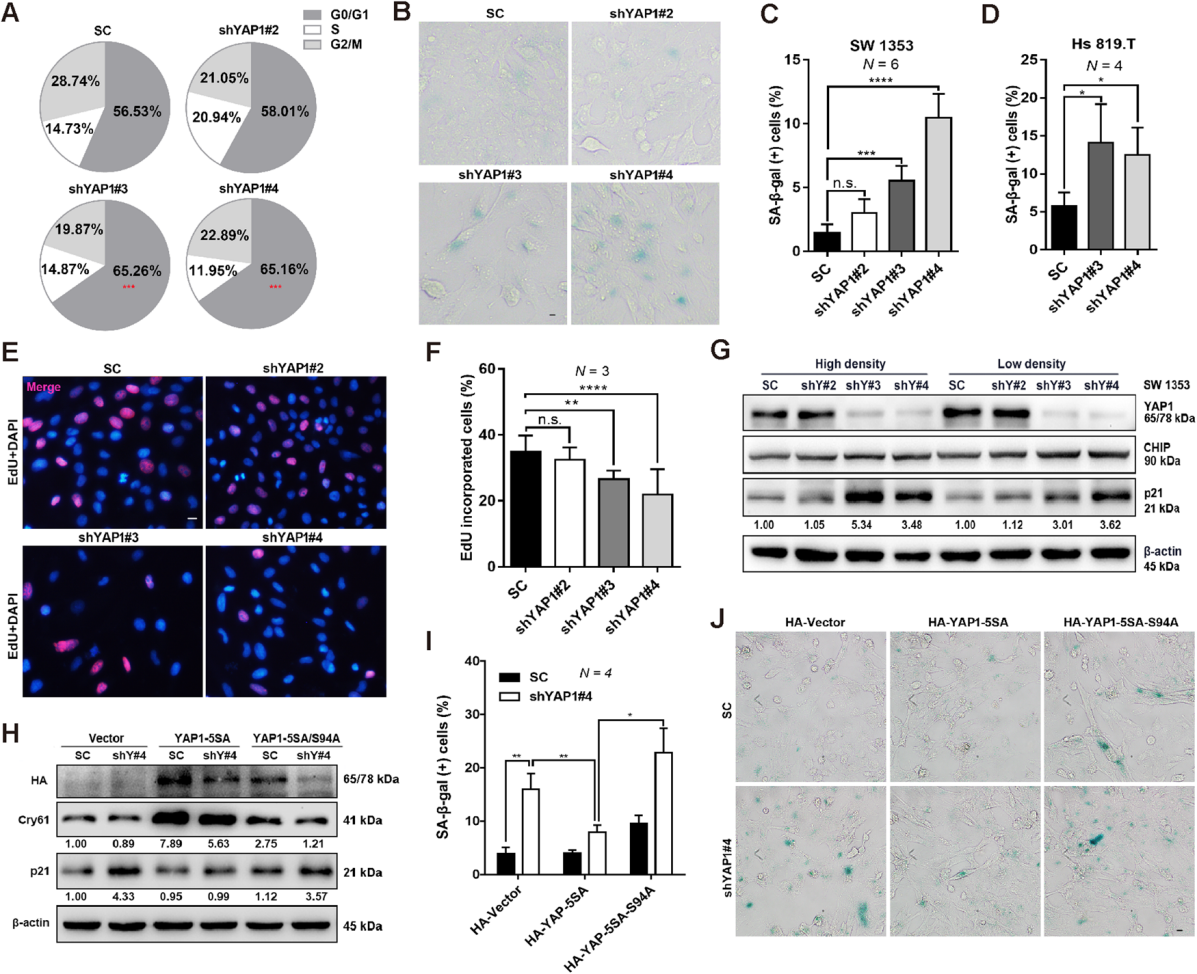
YAP1/TEAD controls cell cycle exit and induces cellular senescence. **A** Cell cycle distribution analysis in the groups of control shRNA and YAP1-specific shRNAs (shYAP#2, #3, #4). **B**, **C** Control shRNA or YAP1-depleted SW 1353 cells were stained with SA-β-gal, and the representative images are shown in (**B**); the percentage of SA-β-gal-positive cells was quantified and shown in (**C**), scale bars: 10 μm. **D** Hs 819.T cells were infected with lentivirus of control shRNA or YAP1-specific shRNAs (shYAP#3, #4), and then the percentage of SA-β-gal-positive cells was quantified. **E**, **F** YAP1 depletion inhibits cell proliferation. Cell proliferation was determined by the EdU incorporation assay (**E**), scale bar: 10 μm. DAPI was used as a nuclear counterstain. The percentage of cells that incorporated EdU was quantified (**F**). **G** Knockdown of YAP1 induces the increase of p21 expression, independent of cell density. Expression of the indicated proteins was determined by IB. **H**–**J** YAP1-TEAD plays a role in regulating CHS cell senescence. control shRNA or YAP1-depleted SW 1353 cells were transiently transfected with HA-vector (Vector), HA-YAP1-5SA (YAP1-5SA), or HA-YAP1-5SA/S94A (YAP1-5SA/S94A) for 72 h, and then the protein levels of HA, Cyr61, and p21 were determined by IB (**H**). The percentage of SA-β-gal-positive cells was quantified (**I**), and representative images are shown in (**J**); scale bars: 10 μm. Data are presented as the mean ± SD of at least three independent experiments in (**A**, **C**, **D**, **F**, **I**). One-way ANOVA followed by Dunnett’s test was applied for (**A**, **C**, **D**, **F**). Two-way ANOVA followed by Tukey’s test for (**I**). n.s. nonsignificant, *P < 0.05, **P < 0.01, ***P < 0.001, ****P < 0.0001.

The control of cancer cell growth by YAP1 can be mediated by modulation of the cell cycle, proliferation, and apoptosis^28^. We thus further explored the roles of YAP1 in the CHS cells. Two validated shYAP1 (i.e., #3 and #4) but not the scramble control (SC) were found to trigger G0/G1 phase cell cycle arrest, as determined by the flow cytometry (Fig. 2A and Fig. S2a). Since YAP1 has been reported to regulate senescence in several cell types^12,29^, we then examined whether YAP1 was required for regulating CHS cell senescence. As expected, YAP1 knockdown significantly increased the percentage of senescence-associated β-galactosidase (SA-β-gal)-positive cells in the two different CHS cell strains, including SW 1353 and Hs 819.T (Fig. 2B–D).

Since chondrocytes tend to undergo senescence when cultured in vitro, we further explored how YAP1 was involved in chondrocyte senescence. AFM confirmed that knockdown of YAP1 increased the chondrocyte’s elastic modulus, a characteristic of senescent cells, at the single-cell level (Fig. S2b). In addition, we demonstrated that YAP1 was essential for CHS cell proliferation, as evidenced by that loss of YAP1 reduced the percentage of EdU-incorporated cells, along with the suppression of c-Myc (Fig. 2E, F and Fig. S2c–e). Although YAP1 plays a crucial role in controlling cell apoptosis^9,30,31^, we found that knockdown of YAP1 dictated CHS underwent senescence rather than apoptosis (Fig. S2f), elucidating a cell-type dependent regulation of YAP1 on CHS.

It has been documented that p21 is one of the primary mediators of cellular senescence^32^; we then examined whether there is any alteration of p21 expression. Notably, loss of YAP1 dramatically upregulated the expression of p21 in the SW 1353 cells, regardless of cell density (Fig. 2G and Fig. S2g). We also found that p16, another primary marker of cellular senescence^19^, was not involved in shYAP1-induced cellular senescence as YAP1 depletion had few effects on the p16 expression (Fig. S2h). Nuclear localization of YAP1 and subsequent interaction with TEAD are critical for initiating downstream signaling of the Hippo/YAP1 axis^26,33^, we therefore asked whether the interaction of YAP1 with TEAD played a role in CHS cell senescence. To this end, we transiently introduced two YAP1 mutants (i.e., HA-YAP1-5SA and HA-YAP1-5SA/S94A) into cells stably expressing SC or shYAP1#4 for further functional study. As expected, overexpression of HA-YAP1-5SA upregulated the expression of its target gene Cyr61 and, reduced p21 expression and p21-induced cellular senescence in cells expressing shYAP1#4 but not SC (Fig. 2H–J). In contrast, the transcriptionally inactive variant HA-YAP1-5SA/S9A had a marginal effect on p21 expression and p21-induced cellular senescence (Fig. 2H–J). Similar results were obtained in primary chondrocytes using these mutants (Fig. S2i–k). Taken together, these results suggest that YAP1 depletion inhibits cell proliferation and induces cellular senescence, and the latter is achieved by the modulation of p21 expression involving the YAP1-TEAD interaction.

### Growth inhibition by YAP1 depletion is negatively correlated with p21 expression in vivo

**Fig. 3:**
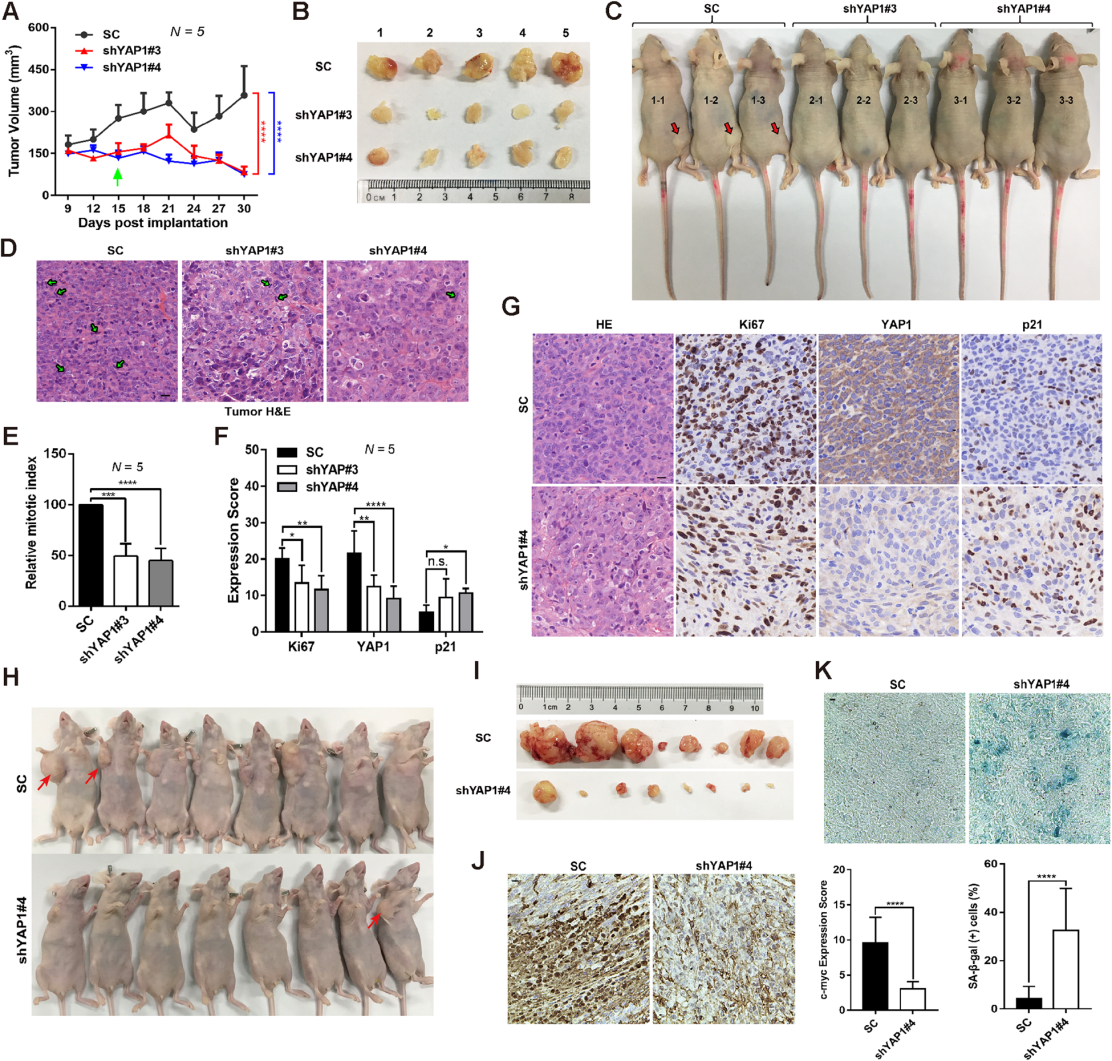
YAP1 coordinates with p21 to control the growth of xenograft tumors. **A**–**C** YAP depletion inhibits xenograft tumor growth. control shRNA or YAP1-depleted SW 1353 cells were subcutaneously inoculated in the flanks of nude mice. Nine days after injection, tumor growth curves were constructed based on the tumor volumes measured every 3 days (**A**); statistical significance was found 15 days post implantation. Ex vivo images of xenograft tumors at 30 days after dissection (**B**); in vivo images of representative nude mice (**C**). **D**, **E** YAP depletion suppresses xenograft tumor cell mitosis. Tumor sections were stained with H & E, arrows indicate mitotic cells (**D**), and the relative mitotic index was quantified (**E**), scale bars: 10 μm. **F**, **G** YAP negatively regulates p21 expression in vivo. Tumor sections of (**B**) were stained with H&E or IHC for Ki67, YAP1, and p21 (**G**); the IHC scores for Ki67, YAP1, and p21 in the mouse tumor sections were measured (**F**), scale bars: 10 μm. **H**, **I** SC and shYAP1#4 stable SW 1353 cells were subcutaneously inoculating into the nude mice at the right flank next to the forelimb for 57 days, the representative in vivo images of nude mice (**H**) and ex vivo xenograft tumors (**I**) were shown. **J** Tumor sections of (**I**) were stained with c-myc (left) and the IHC scores were measured (right). **K** Frozen sections of the fresh tumors were stained with SA-β-gal (above) and the percentage of SA-β-gal-positive cells were determined (below). Data are presented as the mean ± SD of at least three independent experiments in (**A**, **E**, **F**, **K**). One-way ANOVA followed by Dunnett’s test was applied for (**E**, **F**). Two-way ANOVA followed by Tukey’s test for (**A**). Student’s *t*-test for (**K**). n.s.: Nonsignificant, *P < 0.05, **P < 0.01, ***P < 0.001, ****P < 0.0001.

Next, we evaluated the role of YAP1 in xenograft tumor growth in vivo. SW 1353 cells stably expressing SC, shYAP1#3 or shYA1P#4 were inoculated into the flanks of nude mice to establish the xenograft tumors. Loss of YAP1 in the shYAP1#3 and shYAP1#4 groups significantly inhibited the growth of stable cell-derived xenograft tumors (Fig. 3A). At the time of sacrifice, tumor size was measured via both ex vivo and in vivo. The tumor size in mice injected with shYAP1#3- and shYAP1#4-expressing SW 1353 cells was smaller than that in mice that received SC-expressing SW 1353 cells (Fig. 3B, C). In addition, histological assays showed that tumors in mice received shYAP1#3- and shYAP1#4-expressing SW1353 cells exhibited an inhibitory effect on the mitotic index (Fig. 3D, E). Furthermore, loss of YAP1 resulted in an obvious reduction of Ki67-expressing cells (Fig. 3F, G), which is the proliferative marker of multiple cancers. Since a causal relationship between YAP1 and p21 was observed at the cellular level aforementioned, we then investigated the correlation of YAP1 with p21 in this in vivo model. As expected, an increase in p21-positive cells was observed upon YAP1 depletion by shYAP1#4 and shYAP1#3 (Fig. 3F, G). In addition, the suppressive role of YAP1 in CHS tumor growth was further evaluated by subcutaneously inoculating the SC and shYAP1#4 stable cells into the nude mice at the right flank next to the forelimb. As shown in Fig. 3H–K, knockdown of YAP1 did remarkably inhibit the xenograft tumor growth, accompanied by the reduction of c-Myc-stained positive cells and the induction of senescent cells as determined by the SA-β-gal staining using frozen sections. These results together demonstrate that YAP1, via control of p21 expression, is required for xenograft tumor growth in vivo.

### p21 expression is required for the survival of YAP1 depletion-induced senescent cells

**Fig. 4:**
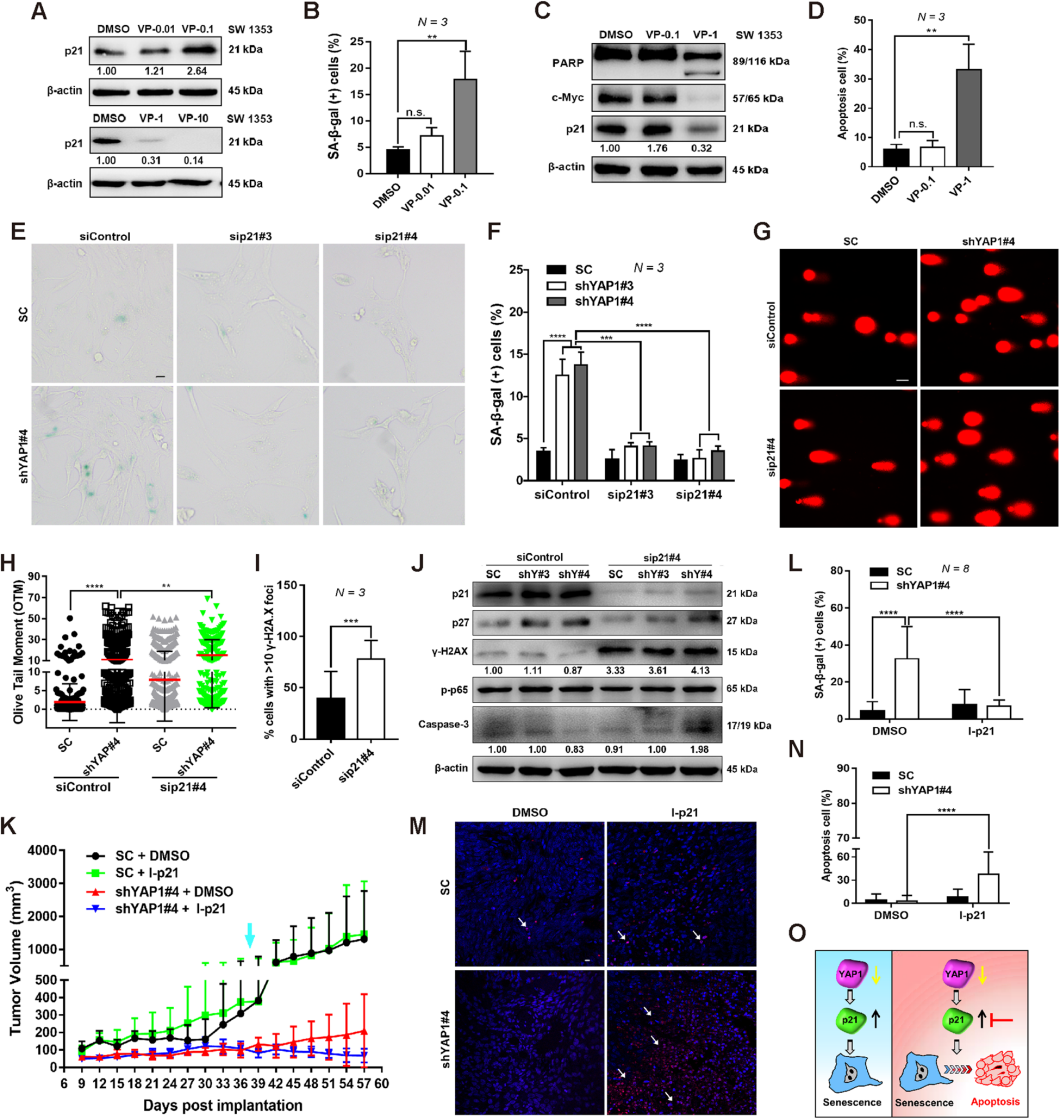
p21 expression maintains the viability of YAP1 depletion-induced senescent cells. **A** IB analysis of p21 expression in SW 1353 cells treated with DMSO or different concentrations of Verteporfin (0.01, 0.1, 1, and 10 μM) for 24 h. **B** The percentage of SA-β-gal-positive cells in the above treatments was quantified. **C** IB analysis of p21, c-Myc, and PARP expression in SW 1353 cells treated with DMSO or different concentrations of Verteporfin (0.1, 1 μM) for 24 h. **D** Flow cytometry analysis of cell apoptosis in the presence or absence of Verteporfin treatments. **E**, **F** Knockdown of p21 abolishes YAP depletion-induced senescence. Control shRNA or YAP1-depleted SW 1353 cells were transfected with p21-specific siRNAs (siControl, sip21#3 and sip21#4) for 72 h followed by SA-β-gal staining (**E**), and SA-β-gal-positive cells were quantified (**F**), scale bars: 20 μm. **G**, **H** p21 knockdown enhances YAP depletion-induced DNA damage. SW 1353 cells stably expressing control shRNA or shYAP1#4 were transfected with p21-specific siRNAs (siControl or sip21#4), and DNA damage was detected by a comet assay (**g**) and analyzed by the OTM (**H**), scale bars: 20 μm. **I** γ-H2AX foci were calculated in shYAP1#4 cells with/without p21 depletion. **J** Knockdown of p21 increases YAP1-depleted cell apoptosis. The expression of the indicated proteins was determined by IB. **K** SC and shYAP1#4 stable SW 1353 cells were subcutaneously inoculating into the nude mice at the right flank next to the forelimb, then the mice 37 days post inoculation (defined with light blue arrow) were treated with p21 inhibitor (I-p21, UC2288) for four times within 7 days, then tumor growth curves were constructed based on the tumor volumes measured every 3 days. **L** Frozen sections of fresh xenograft tumor were stained with SA-β-gal and the percentage of SA-β-gal-positive cells was determined. **M**, **N** Tumor sections of were stained with TUNEL (m, DAPI for nuclear staining) and the TUNEL-stained cells were quantified (**N**). **O** An illustration reveals that targeting p21 in YAP1-depleted cells switches cellular senescence to apoptosis. Data are presented as the mean ± SD of at least three independent experiments in (**B**, **D**, **F**, **H**, **I**, **K**, **L**, **N**). One-way ANOVA followed by Dunnett’s test was applied for (**B**, **D**, **I**). Student’s *t*-test for (**I**). Two-way ANOVA followed by Tukey’s test for (**F**, **K**, **L**, **N**). Mann–Whitney *U* test for (**H**). n.s. nonsignificant, **P < 0.01, ***P < 0.001, ****P < 0.0001.

It has been demonstrated that p21 plays a role in regulating cellular senescence owing to its activity as an inhibitor of cyclin-dependent kinase 2 (CDK2) and CDK4^34^, we therefore examined whether p21 is required for CHS cell senescence using Verteporfin (VP), a small chemical that can inactivate the YAP1 pathway^35^. A lower concentration of VP significantly upregulated p21 expression and increased the percentage of senescent cells (Fig. 4A, B and Fig. S3a), while a higher concentration of VP suppressed the p21 expression, accompanied by pronounced cell apoptosis (Fig. 4C, D).

Recent studies have suggested that persistent expression of p21 may function as an oncogene by promoting the survival of senescent cells^22^, therefore we next investigated whether fine-tuning of p21 expression maintains the viability of senescent cells. Indeed, knockdown of p21 by sip21#3 and sip21#4 remarkably abolished the cell senescence induced by the shYAP1#3 or shYAP1#4 (Fig. 4E, F). We also found a marked increase in DNA damage upon p21 knockdown, as seen from the representative images of the DNA tails and the calculation of the olive tail moment (OTM) (Fig. 4G, H). In addition, we also noted that knockdown of p21 increased γ-H2AX expression, accompanied by the activation of caspase-3-dependent apoptosis (Fig. 4I, J). To further implicate the functional link of YAP1/p21 in CHS, the xenograft tumors established by the SC and shYAP1#4 stable cells were subsequently treated with a p21 inhibitor, UC2288. As shown in Fig. 4K, although non-statistical significance was found upon p21 inhibition (intraperitoneal injection of UC2288) in the shYAP1#4 group, we did found that p21 inhibition visibly decreased the percentage of senescent cells (Fig. 4L). Accordingly, TUNEL staining also revealed that p21 inhibition increased cell apoptosis in the xenograft tumors established by shYAP1#4 (Fig. 4M, N). These findings suggest that the downregulation of p21 expression may switch cellular senescence to apoptosis via the induction of DNA damage in YAP1-depleted cells (Fig. 4O).

### miR-93 is crucial for YAP1-regulated p21 expression and cellular senescence

In response to cellular stimuli, p21 expression is tightly regulated at the transcriptional and posttranslational levels through mechanisms involving RNA stabilization, phosphorylation, and ubiquitination^36^. We thus wondered to elucidate the mechanism(s) underlying YAP1 regulation of p21. In agreement with previous reports, mRNA expression of three YAP1 downstream target genes (i.e., ANK, Cry61, and CTGF) was significantly suppressed upon YAP1 depletion. In contrast, a slight increase in p21/*CDKNA1* mRNA was noted in one of the shRNAs targets of YAP1 (Fig. S3b). p53 is a master regulator of p21 during cellular senescence^37–39^. We then tested whether p21 induction was mediated by the p53, although shYAP1#4 did not induce p53 activation (Fig. S3c). Loss of p53 expression induced by its siRNAs (i.e., sip53#2 and sip53#4) did not abolish the induction of p21 by YAP1 depletion, although p53 knockdown partially attenuated the basal expression of p21 (Fig. S3d). These results indicate that the p21 induction does not occur at p53-dependent transcriptional level.

According to the previous study, Sp1 is an alternative way to positively regulate p21 expression^40^, we next explored the possible transcriptional regulation of p21 by Sp1. Our results herein showed that the expression of Sp1 was downregulated upon YAP1 depletion, excluding the possibility that p21 may be positively regulated by Sp1 (Fig. S3e). Notably, substantial induction of p21 in the cytoplasm was observed upon YAP1 depletion (Fig. S3e), suggesting that the regulation of p21 by YAP1 may occur at the protein level. To confirm this, we used a proteasome inhibitor, MG132. MG132 treatment resulted in the accumulation of p21 in control shRNA; however, it did not further enhance p21 induction in YAP1-depleted cells (Fig. S3f).

**Fig. 5:**
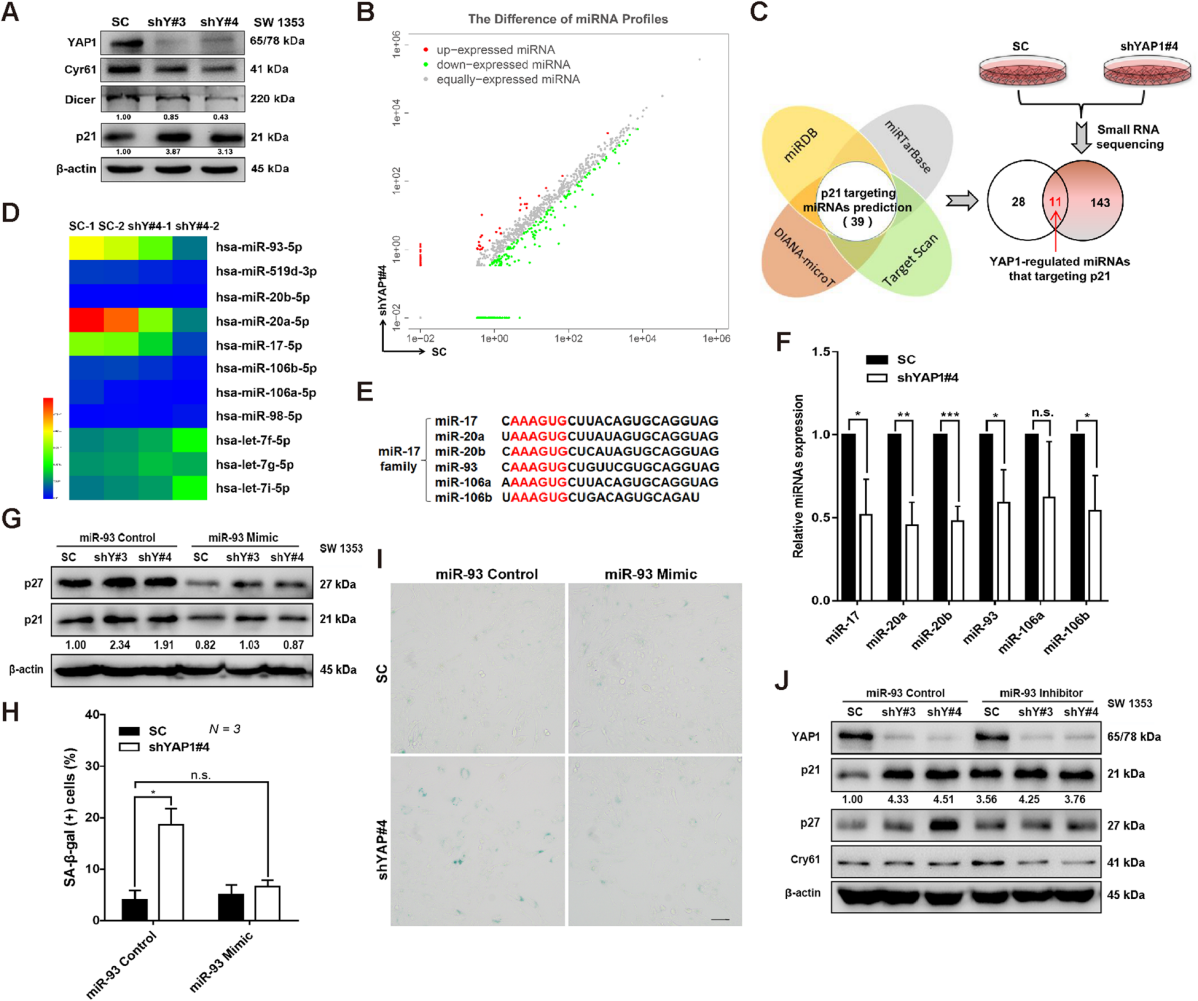
Regulation of p21 by miR-93 is essential for YAP1 depletion-induced cellular senescence. **A** IB analysis of the indicated proteins in control shRNA or YAP1-depleted SW 1353 cells. Cry61 was used as a positive control for the inactivation of YAP1 signaling. **B** A total of 154 miRNAs were found to be up- or downregulated in YAP1-depleted SW 1353 cells by high-throughput small RNA sequencing. **C** Eleven p21-regulating miRNAs were identified in YAP1-depleted SW 1353 cells by overlapping the above sequencing data with the predicted p21-targeting miRNAs with the online tools miRDB, miRTarBase, TargetScan, and DIANA-microT. **D** Heat map of 11 miRNAs regulated by YAP1 in SW 1353 cells. **E** The consensus sequences targeting p21 in the miR-17 family members were aligned. **F** qRT-PCR was used to quantify the expression of miR-17, miR-20a, miR-20b, miR-93, miR-106a, and miR-106b in the indicated cells. **G**–**I** Control shRNA or YAP1-depleted SW 1353 cells were transfected with either miR-93 Control or miR-93 Mimic, and expression of p21 and p27 was analyzed by IB (**G**). The percentage of SA-β-gal-positive cells was quantified (**H**), and representative photos of the senescent cells are shown in (**I**), scale bars: 20 μm. **J** Control shRNA or YAP1-depleted SW 1353 cells were transfected with either miR-93 Control or miR-93 Inhibitor, and expression of the indicated protein was detected by IB. Data are presented as the mean ± SD of at least three independent experiments in (**F**, **H**). Student’s *t*-test for (**F**). Two-way ANOVA followed by Tukey’s test for (**H**). n.s. nonsignificant, **P < 0.01, ***P < 0.001, ****P < 0.0001.

The Hippo-YAP1 signaling pathway is critical for the biogenesis and maturation of numerous miRNAs through modulating key enzymes such as Dicer^41,42^. We then hypothesized that YAP1 regulates p21 expression via the Dicer-miRNA network. As expected, loss of YAP1 function attenuated Dicer expression in the SW 1353 cells (Fig. 5A). Next, we aimed to determine the miRNA profiles that were regulated by the YAP1 using high-throughput small RNA sequencing. A total of 154 miRNAs were up- and downregulated upon YAP1 depletion (Fig. 5B and Fig. S4a). By overlapping the 39 predicted miRNAs that target p21 with the sequencing data, 11 miRNAs were highly regulated by YAP1 in SW 1353 cells (Fig. 5C), and 6 (miR-17, miR-20a, miR-20b, miR-93, miR-106a, miR-106b) out of these 11 belonged to the miR-17 family (Fig. 5D, E). Next, suppression of the expression of miR-17, miR-20a, miR-20b, miR-93, miR-106a, and miR-106b by YAP1 depletion was confirmed by quantitative real-time PCR (Fig. 5F). These findings were supported by a previous study showing that knockout of Dicer also induced robust suppression of miR-17 family members^43^, with the maximal suppressive effect on miR-93 (Fig. S4B).

Furthermore, we explored the possible role of the miR-17 family in YAP1-regulated p21 expression and cellular senescence by taking miR-93 as an example. Strikingly, transfection of the miR-93 mimic further inhibited the expression of p21 (Fig. 5G and Fig. S4c), accompanied by the attenuation of cellular senescence induced by YAP1 depletion (Fig. 5H, I). Interestingly, expression of p21 was increased following transfection with the miR-93 inhibitor in the SC cells, while a comparable expression of p21 was found in cells expressing shYAP#3 and shYAP#4 (Fig. 5J and Fig. S4c), indicating the possible saturation of inhibitory effects of miR-93 on the p21 expression. A corresponding change in the cellular senescence upon miR-93 inhibition was also observed (Fig. S4d, e). Although we cannot exclude the possibility that p21 protein stability might be regulated through other mechanisms, these data collectively suggest that the Dicer1/miR-93 axis plays an important role in regulating p21 expression and subsequent cellular senescence upon YAP1 depletion.

### Sequential targeting of the YAP/p21 axis promotes the elimination of senescent CHS cells

**Fig. 6:**
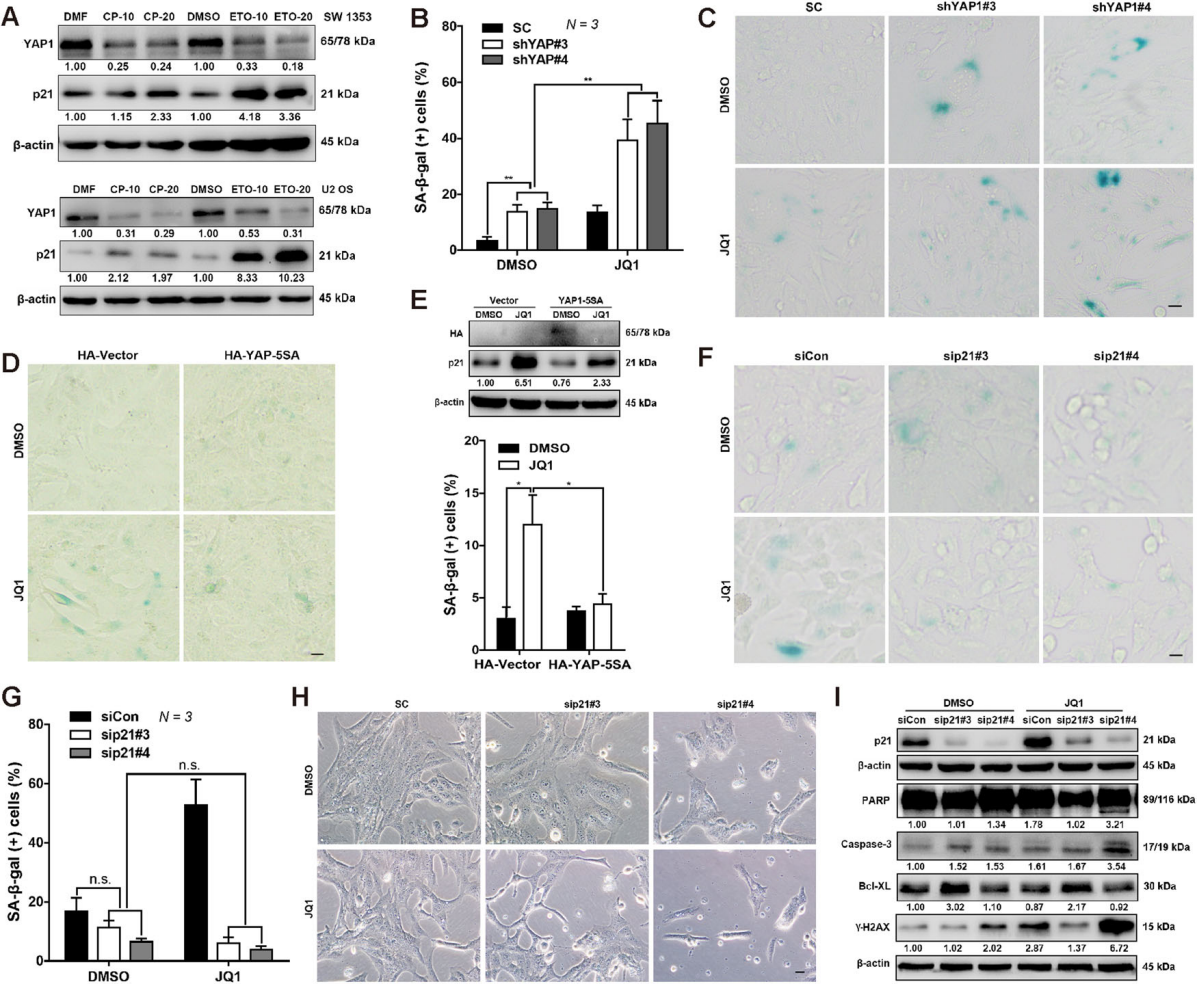
The YAP1/p21 signaling axis is involved in chemotherapy-mediated cell senescence regulation. **A** SW 1353 and U2 OS cells were treated with dimethylformamide (DMF), cisplatin (CP; CP-10: 10 μM, CP-20: 20 μM), dimethyl sulfoxide (DMSO), and etoposide (ETO; ETO-10: 10 μM, ETO-20: 20 μM) for 24 h, and YAP and p21 expression was determined by IB. **B**, **C** YAP1 depletion enhances JQ1-induced senescence. Control shRNA or YAP1-depleted SW 1353 cells were treated with DMSO or JQ1 for 24 h, senescent cells were stained with SA-β-gal and quantified (**B**), and representative images are shown (**C**). Scale bars: 20 μm. **D**, **E** YAP1 overexpression attenuates JQ1-induced senescence. SW 1353 cells were transfected with HA-Vector and HA-YAP-5SA, followed by treatment with DMSO or JQ1 for 24 h, and then the cells were stained with SA-β-gal (**D**), scale bars: 20 μm. The SA-β-gal-positive cells were quantified (**E**, below), and the expression of HA and p21 was confirmed by IB (**E**, above). **F**, **G** Knockdown of p21 abolishes JQ1-induced senescence. SW 1353 cells were transfected with p21-specific siRNAs (sip21#3, sp21#4), followed by DMSO or JQ1 for 24 h. Then, the cells were subjected to SA-β-gal staining (**F**), and the positive cells were quantified (**G**). **H**, **I** Knockdown of p21 transforms JQ1-induced cellular senescence into apoptosis. Cells were treated as in (**F**), and representative cell images are shown in (**H**). Knockdown efficiency and apoptosis-related (PARP, Caspase-3, Bcl-XL) and DNA damage-related (γ-H2AX) protein expression were detected by IB (**I**). Scale bars: 20 μm. Data are presented as the mean ± SD of at least three independent experiments in (**B**, **E**, **G**). Two-way ANOVA followed by Tukey’s test for (**B**, **E**, **G**). n.s. nonsignificant, *P < 0.05, **P < 0.01.

Having established a regulatory role of YAP1 in p21 expression, we wondered to investigate whether the YAP1/p21 axis is implicated in chemotherapy, given that many first-line chemotherapeutic agents trigger cancer cells to undergo senescence^44,45^. As expected, YAP1 expression was downregulated by the first-line chemotherapeutic drugs cisplatin and etoposide, concomitant with the induction of p21 expression in chemotherapy-resistant SW 1353 cells and chemotherapy-sensitive U2 OS cells, in which we had found that knockdown of YAP1 inhibited cell growth and dictated cellular senescence (Fig. 6A and Fig. S5a–c). We have previously demonstrated that SW 1353 cells are sensitive to the epigenetic BET inhibitor JQ1 and that, treatment with JQ1 not only inhibited YAP1 expression but also induced a significant upregulation of p21 expression^46^. Herein, we found that the knockdown of YAP1 further enhanced the cellular senescence induced by JQ1 (Fig. 6B, C). Nevertheless, overexpression of HA-YAP-5SA suppressed the induction of p21 and prevented the cellular senescence induced by JQ1 treatment (Fig. 6D, E). Next, we sought to determine whether p21 also played a role in JQ1-mediated cellular senescence. As expected, loss of p21 by the siRNAs obviously suppressed the cellular senescence triggered by JQ1 in the SW 1353 cells (Fig. 6F, G). Mechanistically, p21 depletion was found to facilitate cell apoptosis by the activation of DNA damage response (DDR) via γ-H2AX and subsequent modulation of the Bcl-XL/Caspase-3 balance (Fig. 6H, I). These results together suggest that the YAP1/p21 axis is required for chemotherapy (e.g., JQ1)-induced cellular senescence.

**Fig. 7:**
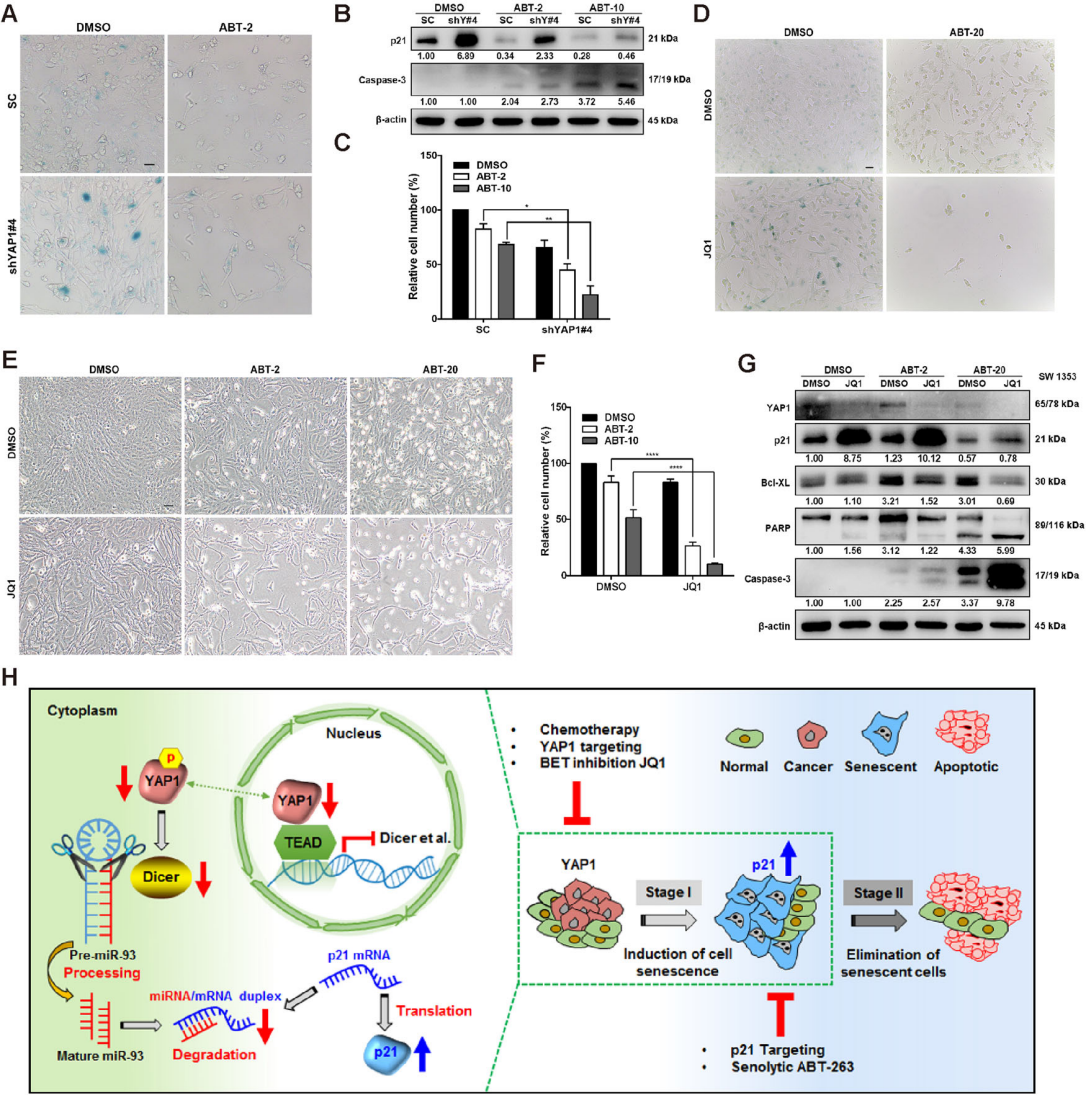
Knockdown of p21 expression enhances DNA damage caused by YAP deletion, induces apoptosis, and weakens cell senescence. **A**–**C** Control shRNA or YAP1-depleted SW 1353 cells were treated with DMSO or ABT (ABT-2: 2 μM, ABT-20: 20 μM) for 24 h, and then the cells were stained with SA-β-gal (scale bar, 20 μm), analyzed by IB, and quantified by cell number quantification. *P < 0.05, **P < 0.01. **D**–**G** SW 1353 cells were treated with DMSO or JQ1 (10 μM) for 24 h, followed by treatment with DMSO or ABT (ABT-2: 2 μM, ABT-20: 20 μM) for another 24 h. Then, the cells were stained with SA-β-gal (**D**, scale bar, 20 μm), and the live cell numbers were counted and quantified (**E**, **F**). The indicated proteins were analyzed by IB (**G**). ****P < 0.0001. **H** Having established a negative correlation between YAP1 and p21-mediated cellular senescence in vitro or in vivo, we then uncovered the underlying regulatory mechanism by which knockdown of YAP1 (via interaction with TEAD) attenuates the expression of Dicer, followed by a decrease in the expression of miR-17 family members, including miR-93, accounting for the induction of p21 and CHS cell senescence (left panel). Based on this, we propose a one-two punch approach to enhance therapeutic efficacy. Briefly, at stage I, induction of p21 expression and cell senescence is achieved by directly targeting YAP1 expression or using chemotherapeutic agents or the BET inhibitor JQ1. At the second stage, directly targeting p21 expression or using the senolytic agent ABT263 is essential for triggering senescent cells to undergo apoptosis, thus enhancing the therapeutic efficacy (right panel). Data are presented as the mean ± SD of at least three independent experiments in (**C**, **F**), followed by two-way ANOVA analysis. *P < 0.05, **P < 0.01, ****P < 0.0001.

Accumulated evidence suggests that a delayed exit of senescent cells induced by first-line chemotherapy supports the tumor environment, which in turn leads to cancer relapse^29,44^. We thus examined whether senolytic molecules, which recently have been developed to suppress cellular senescence by targeting the Bcl-2 family (e.g., Bcl-XL, Bcl-2, and Bcl-W)^15^, can eliminate YAP1 depletion-induced senescent cells. As shown in Fig. 7A–C, the inhibition in senescent cells by ABT263 (a senolytic inhibitor extensively used in clinical trials) was negatively correlated with an increase of cell death in a manner depending on the concentrations of ABT263 in the shYAP1#4 cells (Fig. 7A–C).

Next, we hypothesized that sequential ABT263 treatment may enhance the therapeutic efficacy of JQ1. To test this, senescence was induced by JQ1 for 24 h, followed by the treatment with ABT263 for another 24 h before counting the cell number. As expected, ABT263 treatment significantly suppressed senescent CHS cells, accompanied by a reduction in viable cells (Fig. 7D–F). Furthermore, we found that treatment with ABT263 downregulated the expression of Bcl-XL and p21, while activated the caspase-3 and PARP (Fig. 7G). These results indicate that ABT263 might dictate senescent cells to undergo a caspase-3-dependent apoptotic cell death. Taken together, our results suggest that sequentially targeting the YAP1-p21 axis is a one-two punch approach that leads to more efficient senescent cell eradication (Fig. 7H).

## Discussion

YAP1 is a critical mechanosensitive transcriptional regulator that controls skeletal development and cartilage-related disease^47^. YAP1 is overexpressed in a series of sarcomas, including chondrosarcoma^5,48,49^. However, the functional role of YAP1 in CHS is ill-defined. Our evidence supports the conclusion that YAP1 is critical for modulating the oncogenic phenotypes of CHS. First, expression of YAP1 is negatively associated with the differentiation status of CHS, as poorly differentiated CHS shows higher expression of YAP1. Second, knockdown of YAP1 greatly suppressed cell proliferation, induced cellular senescence in CHS cells, and reduced the growth of xenograft tumors. Of note, the impact of YAP1 on cellular senescence is still controversial, as some studies have demonstrated that depletion of YAP is sufficient to cause senescence-associated phenotypes in mesenchymal stem cells or breast cancer cells^11,50^, while others have found that hyperactivation of YAP1 induces senescence in cultured primary human ovarian surface epithelial cells^13^. These conflicting results of YAP1 in regulating senescence appear to be highly dependent on different cancer settings or cell context-dependent manner. Third, in agreement with previous reports that nuclear YAP1 is critical for cell growth in various cancers, including lung, breast, and prostate cancers, we observed that YAP1 is predominately localized in the nucleus of CHS cells, and that nuclear localization of YAP1 (5SA) induces an oncogenic phenotype in primary chondrocytes and reverses YAP1 depletion-induced senescence in CHS cells. Fourth, It has been demonstrated that nuclear YAP1, via interaction with different transcription factors, including ErbB4, Runx2, NF-Y, mutantp53, p73, and the TEAD family members, is essential for activating many genes related to cell proliferation and antiapoptosis effects^10^. Our results herein also declared a requirement for TEAD in YAP1-regulated cellular senescence. Consistent with the above finding that YAP1 plays a critical role in regulating CHS growth, a recent study also demonstrates that YAP1 signaling is essential for the development of myxoid liposarcoma by driving the *FUS-DDIT3* fusion gene. More importantly, this study reveals that the *FUS-DDIT3* fusion gene physically associates with YAP1 in the nucleus, thus promoting its nuclear localization and elevating transcriptional activity^51^. Although nuclear-localized YAP1 has been demonstrated to cooperate with mutant p53 thus potentiating mutant p53’s gain-of-function activity, our results indirectly suggest that the p53 status might not be the key driver for YAP1-regulated cellular senescence as we had tested wild-type and mutant-type p53 harboring cells. Therefore, unbiased screening of interacting proteins or modifications that mediate YAP1 nuclear localization is of significance in delineating the underlying mechanism of YAP1 implication in CHS.

Two canonical pathways, the p53/p21 and pRb/p16 axes, have been implicated in cellular senescence triggered by the vast majority of stimuli. We have previously found that p21 induction and YAP1 downregulation were concurrently occurred in CHS treated with the BET inhibitor JQ1^46^. In the present study, we were able to demonstrate that p21, but not p53, is fundamental for YAP1-regulated CHS cell senescence. This finding was then generalized to other sarcoma cells including U2 OS cells, as demonstrated by the colony formation and SA-β-gal assay. Since increasing studies have revealed that p21 can be regulated at transcriptional, translational, and posttranslational levels^37^. p21 was first demonstrated to be a downstream target of p53 in vivo in response to DNA-damaging agents^38^. Later, it was found that several other transcription factors, such as Sp1, AP2, and STAT, can also regulate p21 expression^36,37^. In adult frog retinas, YAP1 knockdown was found to cause an upregulation of p21 expression, which involves p53 signaling^52^. In addition to the transcriptional regulation of p21 by YAP1, a recent study has reported that p21 is regulated by SKP2 in a YAP1-dependent manner^28^. Our findings extend current knowledge on the regulatory mechanisms of YAP1 in p21 expression, which is the miR-17 family, miR-93 in particular, in crucial for YAP1-regulated p21 expression and subsequent cellular senescence in CHS. Several evidences have elucidated that YAP/TAZ is involved in miRNA biogenesis and processing through the modulation of Dicer and p72 microprocessor activity^14,42^, thus it will be of interest to examine whether other members or clusters are involved in this regulatory axis in the context of senescence.

Increasing evidence has suggested that p21-mediated cellular senescence functions as a tumor-suppressive mechanism at the early stages of exposure to exogenous and endogenous stresses, including various chemotherapeutic drugs^16^. Whereas, prolonging senescence and p21 expression may elicit its detrimental effects, for instance, promoting cancer recurrence^20,22^. Senescent cells increase the expression and secretion of numerous cytokines, chemokines, and other proteins thus creating a chronic inflammatory microenvironment that favors cancer cell metastasis and tumor recurrence^18^. In addition, recent studies have suggested that the accumulation of senescent cells in tissues and organs drives secondary tumors or cancer relapse^16,17^. Our results demonstrate that the signaling axis of YAP1-p21 is responsive to a variety of chemotherapeutic treatments (cisplatin, etoposide, and JQ1) in different CHS cell lines, suggesting a universal mechanism during the chemotherapy. Therefore, eliminating senescent cells in a precise way enables the maximization of therapeutic efficacy. On the other hand, targeting the YAP1/TEAD expression and their transcriptional activity by BET inhibitors are proposed as promising strategies to overcome chemo-resistance^53,54^.

A recent paper has reported that the inhibition of BET by JQ1 can suppress senescence immune surveillance via its association with super-enhancers^55^. As a result, senescent cells induced by BET inhibition may not be cleared as early as possible, which eventually leads to drug resistance or tumor recurrent. Having observed that the expression of Bcl-XL inhibits the apoptosis of JQ1 herein and previously^46^, we then proposed that inhibition of Bcl-XL may enhance the elimination of senescent cells. Altogether, we demonstrated that sequential targeting of YAP1 and senescence achieves maximum killing efficiency in CHS cells. A working model was also illustrated based on our findings (Fig. 7H), in which, after a first-line therapy (targeting YAP1 or using chemotherapeutic agents or the BET inhibitor), the damaged DNA will dictate cells to undergo senescence; at the second-line therapy, directly targeting p21 expression or using the senolytic agent ABT263 is essential for triggering senescent cells to undergo apoptosis in patients with compromised immune systems that are incapable of clearing senescent cells from the tumor microenvironment.

In summary, our study unveils a previously undescribed role of YAP1 signaling in mediating CHS cell senescence both in vitro and in vivo and demonstrates that the miR-17 family is critical for YAP1-regulated p21 expression and subsequent cellular senescence. Furthermore, we propose a “one-two punch” therapeutic approach by sequentially targeting YAP1 and p21, which maximizes therapeutic efficacy by enhancing the elimination of senescent cells.

## Materials and methods

### Cell culture and reagents

CHON-001, SW 1353, Hs 819.T, and U2 OS cells were obtained from the American Type Culture Collection (ATCC, VA, USA) and authenticated by a polymerase chain reaction of short tandem repeat (STR) sequences, as we described previously^46^. CHON-001, SW 1353, and Hs 819.T cells were maintained in Dulbecco’s modified Eagle’s medium (DMEM, Gibco, cat# C12430500BT, USA). U2 OS cells were cultured in McCoy’s 5 A medium (Life Technologies, cat# 12330031, USA). Primary chondrocytes were isolated from patients who underwent total knee arthroplasty under a signed informed consent form. Patients with bone tumors, tuberculosis, infection, rheumatism, gout were excluded. The cartilage from the femoral condyle was digested with 0.25% trypsin (Thermo Fisher Scientific, cat# 25200072, USA) for 1 h, followed by digestion with type II collagenase (Thermo Fisher Scientific, cat# 17101015, USA) for 4 h at 37 °C. After filtering, the primary chondrocytes were cultured in DMEM. All culture media were supplemented with 10% heat-inactivated fetal bovine serum, 2 mM L-glutamine, and 1% penicillin/streptomycin. JQ1 (HY-13030) and Z-DEVD-FMK (HY-12466) were purchased from MedChem Express (Monmouth Junction, NJ, USA) and diluted with DMSO to a stock concentration of 100 mM. Verteporfin (cat# S1786) was obtained from Selleck Chemicals (Houston, TX, USA), and a working concentration of 1 µM was used to disrupt the YAP1-TEAD interaction. Etoposide (ETO, cat# S1225), cisplatin (CP, cat# S1166), MG132 (cat# S2619), and ABT263 (cat# S1001) were purchased from Selleck Chemicals (Houston, TX, USA). CP was diluted in DMF, and the others were diluted with dimethyl sulfoxide (DMSO) unless indicated otherwise. The same volume of DMSO was added and served as an internal control for treatment with inhibitors. Lipofectamine 2000 transfection reagent was purchased from Invitrogen (Thermo Fisher Scientific, cat# 11668019, USA).

### Cell viability assay

SW 1353 cells were seeded in 96-well plates at a density of 2000 cells/well and then treated with different concentrations of JQ1 for various times. Cell viability was determined by Cell Counting Kit-8 (CCK-8, Beyotime, cat# C0042, China) according to the manufacturer’s instructions. The optical densities (ODs) were measured at a 450 mm wavelength with a multimode microplate reader (TriStar LB 941, Berthold, Germany).

### Cell cycle analysis

Stable cells were cultured for 3 days before harvesting. Cell cycle progression was examined using a cell cycle detection kit (KeyGen Biotech, KGA512, China) by C6 flow cytometer (BD Biosciences, CA, USA), and the relative distributions of the G1, S, and G2 phases were analyzed by FlowJo 7.6.5 software (Tree Star Inc., Ashland, OR, USA).

### Cell mechanical property evaluation

The elastic modulus, adhesion, and morphology of living cells were determined in contact mode by atomic force microscopy (AFM, BioScope Catalyst, Bruker Instruments, Germany). First, the cells were fixed with 4% paraformaldehyde (Beyotime, cat# P0099, China) for 15 min, washed three times with phosphate-buffered saline (PBS) for 3–5 min and immersed in PBS. The AFM force volume model was employed to measure the adhesion force and elastic modulus. The FV image was captured in the cytoplasmic region with a scan size of 5 × 5 μm, and more than 1000 force curves for each separate experiment were analyzed. The basic scan parameters were as follows: the ramp size was set as 1.5 μm, the forward and retraction velocity was 500 nm/s, and the spring constant of the AFM tip was 0.14380 N/m. Force curves were fit to the Sneddon model to estimate the elastic modulus. The experimental force-indentation curves were fitted with the formula

$$F = \frac{2}{\pi }\frac{E}{{\left( {1 - \nu } \right)^2}}{\mathrm{tan}}\left( \alpha \right)\delta ^2$$

where F, E, δ, α, and ν are the loading force, elastic modulus, depth of indentation, tip half angle of 18°, and Poisson’s ratio of 0.5, respectively.

### Ethynyl deoxyuridine (EdU) incorporation assay

To detect DNA synthesis, cells were seeded in a 12-well plate at a density of 5 × 10^4^ cells/well and cultured for 72 h. Then the cells were incubated with 20 µM EdU pretreatment solution for another 2 h, followed by fixation with 4% paraformaldehyde. Then, EdU staining and Hoechst 33342 staining were performed using the Cell-Light EdU Apollo 567 In Vitro Imaging Kit (EdU, RiboBio, cat# C10310-1, China) according to the instructions. The ratio of EdU-positive cells/total cells (Hoechst 33342-positive cells) was calculated from ten random fields from three independent experiments.

### Colony formation assay

Cells were seeded into 6-well plates at a density of 1000 cells per well and allowed to adhere in complete DMEM for eight days. Then, the colonies were washed with PBS and stained with crystal violet solution (Beyotime, cat# C0121, China). The relative number of colonies that contained more than 50 cells was counted under the SZ760 series microscope (Chongqing Optec Instrument Co., Ltd., China). Each experiment was performed in duplicate and repeated three times.

### Quantitative real-time reverse-transcriptase polymerase chain (qRT-PCR) reaction

Total RNA was isolated using the PureLink RNA Mini Kit (Thermo Fisher Scientific, cat# 12183018 A, USA). The RNA concentration was determined with a NanoDrop system (NanoDrop Technologies Inc., USA) and then reverse-transcribed using the High Capacity cDNA Reverse Transcription Kit (Applied Biosystems, cat# 4368814, USA). Specific primer sets for amplifying target genes were listed in Table S2. Target gene expression was calculated based on the ∆∆Ct method and normalized to glyceraldehyde-3-phosphate dehydrogenase (GAPDH) as a reference gene. For miRNA quantification, the miRcute miRNA Isolation Kit (Tiangen Biotech, DP501, China) was used to isolate the miRNA according to the manufacturer’s instructions. One hundred nanograms of miRNA was used for reverse transcription according to the miRcute Plus miRNA First-Strand cDNA Kit (Tiangen Biotech, KR211, China). The expression level of miRNA was quantified with the miRcute Plus miRNA qPCR Kit (Tiangen Biotech, FP411, China). The specific forward primers were listed in Table S5. The 2-ΔΔCt method was used to determine the miRNA expression level. All PCRs were performed in triplicate on the DNA engine CFX96 Real-Time PCR amplification system (BIO-RAD, CFX96 Touch, Germany).

### Preparation of nuclear and cytoplasmic protein extracts

A cell fractionation assay was conducted using an NE-PER Nuclear and Cytoplasmic Extraction Kit (Thermo Fisher Scientific, cat# 78835, MA, USA) according to the manufacturer’s instructions. Briefly, cells were harvested, washed with PBS and then incubated with ice-cold cytoplasmic extraction reagents (CER) I for 10 min. After incubation, the CER II extraction reagent was added, followed by centrifugation for 5 min at maximum speed in a microcentrifuge (Heal Force, Neofuge 13R, China) (16,000 × *g*). The supernatant (cytoplasmic extract) was transferred to a clean prechilled tube, while the insoluble (pellet) fraction was resuspended with nuclear extraction reagents (NER) and incubated for a total of 40 min on ice. The supernatant after centrifugation at 16,000 × *g* for 10 min was then collected as the nuclear extract.

### Immunoblots

Total protein lysate was prepared as we described previously^56^. Cytoplasmic and nuclear proteins were extracted using a nuclear and cytoplasmic extraction kit (Thermo Fisher Scientific Inc.) according to the manufacturer’s instructions. Briefly, aliquots of each sample containing 20~50 µg of protein were separated by electrophoresis in 8–15% sodium dodecyl sulfate-polyacrylamide gel electrophoresis (SDS-PAGE) gels (Beyotime, cat# P0012A, China), and transferred to polyvinylidene fluoride membranes (PVDF, PALL, cat# BSP0161, MA, USA). After blocking with non-fat dry milk for 1 h at room temperature, the membranes were probed with primary antibodies obtained from Cell Signaling Technology (CST, MA, USA) or Abcam (UK): anti-YAP1 (CST, cat# 14074, USA, 1:1000), anti-HA (CST, cat# 2367, USA, 1:1000), anti-Cyr61 (CST, cat# 14479, USA, 1:1000), anti-Sp1 (CST, cat# 9389, USA, 1:1000), anti-p16 (Abcam, cat# ab51243, UK, 1:1000), anti-Dicer (CST, cat# 5362, USA, 1:1000), anti-γ-H2A.X (CST, cat# 9718, 1:1000), anti-p-p65 (CST, cat# 3033, USA, 1:1000), anti-caspase-3 (CST, cat# 9664, USA, 1:1000), anti-PARP (CST, cat# 9532, USA, 1:1000), anti-TAZ (CST, cat# 8418, USA, 1:1000), anti-β-actin (CST, cat# 3700, USA, 1:2000), anti-p53 (CST, cat# 9282, USA, 1:1000), anti-c-Myc (CST, cat# 13987, USA, 1:1000), anti-Bcl-xL (CST, cat# 2764, USA, 1:1000), anti-p21 (CST, cat# 2947, USA, 1:1000), anti-β-Tubulin (CST, cat# 2128, USA, 1:5000), and anti-Lamin-A/C (CST, cat# 2032, USA, 1:5000). Next, the membranes were incubated with HRP-linked anti-mouse IgG (CST, cat# 7076; 1:1000) or HRP-linked anti-rabbit IgG (CST, cat# 7074; 1:1000) secondary antibodies (Cell Signaling Technology, USA), and the blots were detected using Clarity Western ECL Substrate (BIO-RAD, cat# 1705061, Germany). The images were captured using a Tanon 5200 Luminescent Imaging Workstation (Tanon, China), and analyzed using ImageJ software (National Institutes of Health, Bethesda, MD, USA).

### Plasmid construction

YAP1 cDNA was gifted by Prof. Ximei Wu (Zhejiang University) and was subcloned in-frame into the HA-CMV vector using two enzymatic sites, BglII and NotI. A series of constructs encoding HA-YAP1-5SA, HA-YAP1-5SA-S94A, and mutants were generated by using ligation PCR, as we described previously^57^. Primers used for HA-YAP1-5SA (five mutated phosphorylation sites) and HA-YAP1-5SA-S94A are listed in Table S4. All of the constructs were verified by sequencing and checked for expression.

### Single-cell gel electrophoresis

The DNA Damage Detection Kit was purchased from KeyGen Biotech (KGA240-50, China), and the experiments were conducted according to the manufacturer’s instruction, with minor revision as we described previously^58^. In brief, the cells were resuspended in PBS and allowed for solidification at 4 °C for 20 min on slides. The comet slides were then immersed in prechilled lysis buffer from the kit and washed twice with PBS. To assess DNA double-strand breaks (DSBs), migration was performed under alkaline conditions (pH = 12.3). After migration, the slides were washed in 0.4 mM Tris-HCl buffer (pH = 7.5) at 4 °C three times. Then, the slides were stained with propidium iodide (PI) in a dark box. The damaged DNA was detected by a Leica DMi8 fluorescence microscope (Leica Microsystems, Germany) at a 515~560 nm excitation wavelength. The OTM was analyzed by OpenComet software^59^.

### RNA interference

siRNAs targeting four different sequences of p53 and p21 (Gene Pharma, China) were used to perform the knockdown experiments. The four different sequences were listed in Table S2. For transient knockdown experiments, cells were seeded into a 6-well plate (Corning, Costar, cat# 3516, USA) for 24 h, and then the siRNAs were transfected with Lipofectamine 2000 (Thermo Fisher Scientific, cat# 11668019, USA) for 4 h in 1 mL of FBS free medium. Then, a complete medium was added, and the cells were cultured for another 72 h.

### shRNA construction and establishment of stable shYAP1 cell lines

Lentivirus particles expressing shYAP1 were produced by GenePharma Co., Ltd. (Shanghai, China). Briefly, a series of YAP1 targeting sequences were selected and cloned into a lentivirus-based RNAi system (pGLV3/H1/GFP + Puro Vector, GenePharma). shRNA target sequences were listed in Table S1. The lentiviral shRNA-expression plasmids (SC or shY#4) were transfected with the packaging plasmids into 293T cells for lentivirus generation. Supernatants containing lentivirus were then harvested and concentrated. SW 1353 cells were then infected by applying viral supernatant (multiplicity of infection, MOI = 20) in 10 mL of complete medium for 24 h, and the stable cell lines were selected using 2 μg/mL puromycin for 3 days for two passages. Puromycin (1 μg/mL) was used to maintain the cells.

### High-throughput sequencing and bioinformatics analysis

High-throughput sequencing was completed by RiboBio Co. Ltd. (Guangzhou, China). The operation procedure can be briefly described as follows. Total RNA was extracted, purified and confirmed for quantity and integrity, and then 1 μg of total RNA from each sample was used to prepare small RNA libraries with the NEBNext® Multiplex Small RNA Library Prep Set for Illumina (NEB, USA) according to the manufacturer’s instructions. The libraries were used for sequencing on the Illumina HiSeqTM 2500 sequencing platform (Illumina, San Diego, CA, USA). Bioinformatics analysis of raw sequence data was conducted, and the ensemble transcript database was used to annotate the results. TargetScan (http://www.targetscan.org/), miRDB (http://mirdb.org/), miRWalk (http://mirwalk.umm.uni-heidelberg.de) and miRbase (http://www.mirbase.org/) were applied to predict the target genes of the selected miRNAs.

### miRNA transfection

The miR-93 mimic control, hsa-miR-93 mimic (cat# miR10000093-1-5), miR-93 inhibitor control, and hsa-miR-93 inhibitor (cat# miR20000093-1-5) were purchased from RiboBio (Guangzhou, China). For miRNA transfection, SW 1353 cells were seeded into a 6-well plate for 24 h, and 5 μL of miR-93 mimic or 2.5 μL of miR-93 inhibitor was transfected with 6 μL of Lipofectamine 2000 for 4 h in 1 mL of FBS free medium. Then, a complete medium was added and cells were cultured for another 72 h.

### Senescence-associated β-galactosidase staining

Senescence-associated β-galactosidase (SA-β-gal) staining was performed using a commercial kit (Beyotime, C0602, China) according to the manufacturer’s instructions. Briefly, cells were grown in 6-well plates at a density of 1 × 10^5^/well and tissue sections were made from frozen sections of tumor tissue peeled from nude mice. After treatment, the cells or tissue sections were fixed with β-galactosidase fixative solution for 15 min, followed by staining with SA-β-gal staining solution at pH 6.0 and 37 °C for 6–24 h (depending on the cell line). The senescent cells were observed and captured with an Olympus IX51 (Olympus, Tokyo, Japan). The percentage of SA-β-gal-positive cells was quantified from at least three random fields per sample (a minimum of 300 cells) and calculated as: (β-gal-positive cells/total cells in a field) × 100%.

### Tissue microarray and immunohistochemistry (IHC)

OS803 chondrosarcoma tissue microarray was purchased from US Biomax, Inc. The staining was conducted by pathologists, and the scoring was performed as previously reported^60^. Briefly, slides were deparaffinized in xylene and rehydrated through a graded alcohol series before endogenous peroxidase activity was blocked with 3% H_2_O_2_ in methanol. Antigen retrieval was achieved by incubating the slides in Citrate buffer at 60 °C for 20 min, followed by blocking with goat serum to avoid nonspecific binding. The primary anti-YAP1 antibody (Novus Biologicals, cat# NB110-58358, USA, 1:100), c-myc (Abcam, cat# ab32072, UK, 1:100), Ki67 (Servicebio, cat# GB111499, China, 1:200), p21 (CST, cat# 2947, USA, 1:50) were added overnight at 4 °C. Then the samples were incubated with HRP-conjugated secondary antibodies (CST, cat# 7074, 1:100) at room temperature for another 1 h, followed by counterstaining with hematoxylin. All images were acquired using a light microscope (Leica, 390335, Germany). The semi-quantification of YAP1 expression was performed as follow: The intensity was scored as 0 (no expression), 1 (low expression), 2 (moderate expression), and 3 (high expression), and the percentage of cells showing the expression was scored ranging from 0 to 10 with 10 as the highest percentage (100%). The expression score for cytoplasmic- and nuclear-localized YAP1 was respectively determined by the intensity score times the percentage (0–30), and the total expression score is the sum of the cytoplasmic and nuclear expression scores (0–60).

### Immunofluorescence (IF)

Cells were seeded onto coverslips precoated with poly-L-lysine and then cultured for 72 h. The cells were washed with PBS and then fixed with 4% polyformaldehyde and permeabilized with 0.2% Triton X-100. After blocking with 5% nonfat milk for 30 min, the cells were incubated with antibodies against YAP1 (CST, cat# D8H1X, 1:100, USA), 53BP1 (CST, cat# E7N5D, 1:200, USA), γ-H2AX (CST cat# 20E3, 1:100, USA) and F-actin (Invitrogen, cat# MA1-80729, 1:100, USA) for 2 h at RT. After three washes with TBST, the cells were incubated with anti-rabbit IgG (H + L), F(ab’)2 Fragment Alexa Fluor® 488 Conjugate (CST, cat# 4412, 1:100, USA), anti-rabbit IgG (H + L), F(ab’)2 Fragment Alexa Fluor® 555 Conjugate (CST, cat# 4413, 1:100 USA) or anti-mouse IgG (H + L), F(ab’)2 Fragment Alexa Fluor® 555 Conjugate (CST, cat# 4409, 1:100, USA) for 1 h in the dark and then washed three times as described above. Nuclei were counterstained with 4′,6-diamidino-2-phenylindole (DAPI, Sigma, cat# 32670, USA). The images were captured under a confocal microscope (Carl Zeiss, Zeiss LSM 700, Germany) with a ×63 objective lens. The percentage of foci of 53BP1 and γ-H2AX was automatically counted with ImageJ using the ITCN plugin in 10 fields for a total of more than 100 nuclei.

### Xenograft tumor growth in nude mice

The animal experiments were approved by the Jinan University Animal Care and Use Committee and conformed to the “Guide for the Care and Use of Laboratory Animals” of the National Institute of Health in China. Five- to six-week-old BALB/c nu/nu mice were purchased from the Model Animal Research Center of Nanjing University (Nanjing China). SW 1353 cells infected with shYAP1 (shYAP#3, shYAP#4) or SC were harvested and then washed with PBS three times. Then, the cells were resuspended at 1 × 10^7^ cells/mL in prechilled PBS-diluted Matrigel (BD Biosciences, cat# 354248, USA, 1:1 by volume). Then, 0.2 mL of the resuspended cells were randomly injected subcutaneously into the flanks of mice and allowed to grow for ~1 month. At the end of the experiments, the tumors were resected (*N* = 5), formalin fixed, and paraffin embedded. For p21 inhibition experiments, xenograft tumors were established by subcutaneously inoculating the SC and shYAP1#4 stable cells into the nude mice at the right flank next to the forelimb. Each group of nude mice transplanted with the SW1353 cells was randomly divided into DMSO and UC2288 (p21 inhibitor; MERCK, cat# 5.32813.0001, USA) treated group. UC2288 was dissolved in DMSO with a concentration of 5 mg/mL and was intraperitoneal injected with 10 mg/kg for four times within 7 days. Tumor growth was monitored every 3 days, and the tumor volume was calculated using 1/2 × L × W × H. All of the sections (4 µm) were cut and stained with hematoxylin and eosin. Frozen section was prepared for cellular senescence analysis. For IHC staining, sections were washed with PBS, developed using a DAB kit (CST, cat# 8059, USA) and then counterstained with hematoxylin. All of the IHC results were scanned by Pannoramic MIDI (3DHISTECH, Hungary). For the determination of cell apoptosis in tissues, TUNEL Apoptosis Detection kit (Yeasen, cat# 40308, China) was used according to the manufacturer’s instructions.

### Statistical analysis

Statistical analysis was performed using GraphPad Prism (GraphPad Software, San Diego, CA, USA). All of the results are shown as the mean ± standard deviation (SD). Data were reported as biological replicates, with technical replicates indicated in the Figure legends. The student’s *t*-test was used to compare values of test and control samples. One-way analysis of variance (ANOVA) was used for multiple group comparison, followed by Dunnett’s test. Two-way ANOVA was applied to compare the means of two independent variables, followed by Tukey’s test. Significance levels of *P* < 0.05, < 0.01, < 0.001, < 0.0001 were denoted in graphs by different asterisks.

## Supplementary information

SUPPLEMENTAL MATERIAL
