# Supplementary material for: Sequential targeting of YAP1 and p21 enhances the elimination of senescent cells induced by the BET inhibitor JQ1
Source: Cell Death Dis. 2021 Jan 25;12(1):121. doi: 10.1038/s41419-021-03416-1 (PMC7835383; doi:10.1038/s41419-021-03416-1)
Supplement: Supplementary file 1 — SUPPLEMENTAL MATERIAL [file 41419_2021_3416_MOESM1_ESM.docx]

**Supplemental materials**

**Materials and methods**

**Sequences using for shRNA, the real-time PCR, siRNAs, and plasmids constructions**

**Table S1. Sequences of the indicated shRNAs**

| **Target** | **Sequence** |
| --- | --- |
| **ShControl** | 5′-**TTCTCCGAACGTGTCACGT**-3′ |
| shYAP1#1 | 5′-CATAAGAACAAGACCACCTCT-3′ |
| shYAP1#2 | 5′-CACAGGCAATGCGGAATATCA-3′ |
| shYAP1#3 | 5′-CCTTAACAGTGGCACCTATCA-3′ |
| shYAP1#4 | 5′-GGTGATACTATCAACCAAAGC-3′ |

**Table S2. List of the primers used for the real-time PCR**

| **Genes** | **Forward** | **Reverse** |
| --- | --- | --- |
| YAP1 | 5′-GCCATGTTGTTGTCTGATCG-3′ | 5′-CACAGCTCAGCATCTTCGAC-3′ |
| P21 | 5′-AGGGGACAGCAGAGGAAGA-3′ | 5′-GGCGTTTGGAGTGGTAGAAAT-3′ |
| ANKRD1 | 5′-TAGGCACATCCACAGGTTCC-3′ | 5′-GAAACAACGAGAGGCAGAGC-3′ |
| Cyr61 | 5′-TACACTGGCTGTCCACAAGG-3′ | 5′-CTTCATGGTCCCAGTGCTC-3′ |
| CTGF | 5′-TTGGGAGTACGGATGCACTT-3′ | 5′-TACCAATGACAACGCCTCCT-3′ |
| β-actin | 5′TGACAGGATGCAGAAGGAGA-3′ | 5′GCTGGAAGGTGGACAGTGAG-3′ |

**Table S3. List of the siRNAs sequences targeting YAP1, p53, p21and TAZ**

| **Genes** | **Forward** | **Reverse** |
| --- | --- | --- |
| YAP1#1 | 5′-GCAUCUUCGACAGUCUUCUTT-3′ | 5′-AGAAGACUGUCGAAGAUGCTT-3′ |
| YAP1#2 | 5′-GGUCAGAGAUACUUCUUAATT-3′ | 5′-UUAAGAAGUAUCUCUGACCTT-3′ |
| YAP1#3 | 5′-GACGACCAAUAGCUCAGAUTT-3′ | 5′-AUCUGAGCUAUUGGUCGUCTT-3′ |
| YAP1#4 | 5′-GGUGAUACUAUCAACCAAATT-3′ | 5′-UUUGGUUGAUAGUAUCACCTT-3′ |
| p53#1 | 5′-CCGGACGAUAUUGAACAAUTT-3′ | 5′-AUUGUUCAAUAUCGUCCGGTT-3′ |
| p53#2 | 5′-GUACCACCAUCCACUACAATT-3′ | 5′-UUGUAGUGGAUGGUGGUACTT-3′ |
| p53#3 | 5′-GUAAUCUACUGGGACGGAATT-3′ | 5′-UUCCGUCCCAGUAGAUUACTT-3′ |
| p53#4 | 5′-GUACCACCAUCCACUACAATT-3′ | 5′-UUGUAGUGGAUGGUGGUACTT-3′ |
| p21#1 | 5′-GAUGGAACUUCGACUUUGUTT-3′ | 5′-ACAAAGUCGAAGUUCCAUCTT-3′ |
| p21#2 | 5′-CCUCUGGCAUUAGAAUUAUTT-3′ | 5′-AUAAUUCUAAUGCCAGAGGTT-3′ |
| p21#3 | 5′-CAGGCGGUUAUGAAAUUCATT-3′ | 5′-UGAAUUUCAUAACCGCCUGTT-3′ |
| p21#4 | 5′-GGAACAAGGAGUCAGACAUTT-3′ | 5′-AUGUCUGACUCCUUGUUCCTT-3′ |
| TAZ | 5′- GCCACAUCUGGAACCUGAATT -3′ | 5′- UUCAGGUUCCAGAUGUGGCTT -3′ |
| NC | 5′-UUCUCCGAACGUGUCACGUTT-3′ | 5′-ACGUGACACGUUCGGAGAATT-3′ |

**Table S4. List of the primers used for the plasmids construction**

| **Genes** | **Forward** | **Reverse** |
| --- | --- | --- |
| YAP1 | 5′-GAAGATCTCTATGGATCCCGGGCAGCA-3′ | 5′-TAAAGCGGCCGCCTATAACCATGTAAGAAAGCTTTC-3′ |
| YAP1-S61A | 5′-GTCCGCGGGGACGCGGAGACCGACCT-3′ | 5′-AGGTCGGTCTCCGCGTCCCCGCGGAC-3′ |
| YAP1-S94A | 5′-GGAAGCTGCCCGACGCCTTCTTCAAGCCG-3′ | 5′-GCGGCTTGAAGAAGGCGTCGGGCAGCTTCC-3′ |
| YAP1-S109A | 5′-ACTCCCGACAGGCCGCTACTGATCGAGGCAC-3′ | 5′-GTGCCTGCATCAGTAGCGGCCTGTCGGGAGT-3′ |
| YAP1-S127A | 5′-AGCATGTTCGAGCTCATGCCTCTCCAGCTTCTCTG-3′ | 5′-CAGAGAAGCTGGAGAGGCATGAGCTCGAACATGC-3′ |
| YAP1-S164A | 5′-CTCAGCATCTTCGACAGTCTGCTTTTGAGATACCTGATGATG-3′ | 5′-CATCATCAGGTATCTCAAAAGCAGACTGTCGAAGATGCTGAG-3′ |
| YAP1-S381A | 5′-CCTATCACTCTCGAGATGAGGCTACAGACAGTGGACTAAGC-3′ | 5′-GCTTAGTCCACTGTCTGTAGCCTCATCTCGAGAGTGATAGG-3′ |

**Table S5. List of the sequences used for qPCR analysis of miRNAs**

| **Target** | **Sequence** |
| --- | --- |
| hsa-miR-17-5p | GUCAGAAUAAUGUCAAAGUGCUUACAGUGCAGGUAGUGAUAUGUGCAUCUACUGCAGUGAAGGCACUUGUAGCAUUAUGGUGAC |
| hsa-mir-20a-5p | GUAGCACUAAAGUGCUUAUAGUGCAGGUAGUGUUUAGUUAUCUACUGCAUUAUGAGCACUUAAAGUACUGC |
| hsa-mir-20b-5p | AGUACCAAAGUGCUCAUAGUGCAGGUAGUUUUGGCAUGACUCUACUGUAGUAUGGGCACUUCCAGUACU |
| hsa-mir-93-5p | CUGGGGGCUCCAAAGUGCUGUUCGUGCAGGUAGUGUGAUUACCCAACCUACUGCUGAGCUAGCACUUCCCGAGCCCCCGG |
| hsa-mir-106a-5p | CCUUGGCCAUGUAAAAGUGCUUACAGUGCAGGUAGCUUUUUGAGAUCUACUGCAAUGUAAGCACUUCUUACAUUACCAUGG |
| hsa-mir-106b-5p | CCUGCCGGGGCUAAAGUGCUGACAGUGCAGAUAGUGGUCCUCUCCGUGCUACCGCACUGUGGGUACUUGCUGCUCCAGCAGG |

**Results**


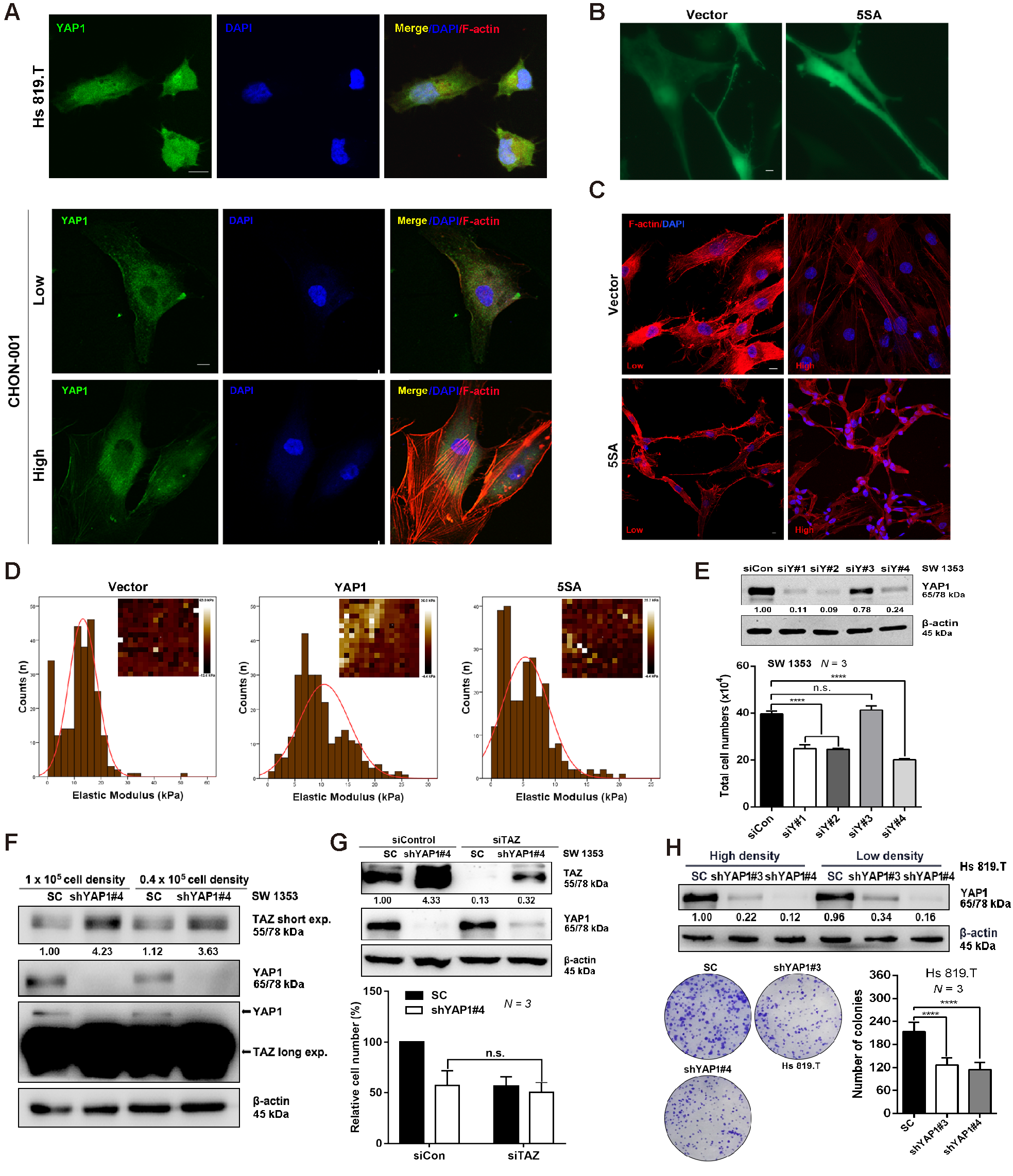


**Fig. S1. Knockdown of YAP1 inhibits the growth of CHS cells. a** Representative IF images of YAP1 localization (green) in Hs 819.T (above) and CHON-001 (below) cells. DAPI (blue) indicates the nucleus, while F-actin (red) defines the cytoskeleton. Scale bars: 10 μm. Low indicated a cell density at 4×10^4^ cells/well, while High indicated a cell density at 1×10^5^ cells/well. **b** Representative IF images of CHON-001 cells infected with lentivirus-delivered HA-vector (Vector) or HA-YAP1-5SA (5SA). Scale bars: 10 μm. **c** Representative IF images of the cytoskeleton with F-actin staining in primary chondrocytes infected with lentivirus-delivered HA-vector (Vector) or HA-YAP1-5SA (5SA). Scale bars: 10 μm. Low indicated a cell density at 4×10^4^ cells/well, while High indicated a cell density at 1×10^5^ cells/well. **d** The elastic modulus of primary chondrocytes analyzed by AFM. **e** SW 1353 cells were transiently transfected with either siControl (siCon) or YAP1-specific siRNAs (siY#1, #2, #3, #4), and YAP1 expression was detected by IB (above). The total cell number with or without YAP1 knockdown was quantified (below). **f** IB analysis of expression of the indicated proteins in SW 1353 cells stably expressing control shRNA or YAP1#4 at different cell densities. **g** Determination of cell growth upon YAP1/TAZ double-knockdown in the SW 1353 cells. The knockdown efficiency was confirmed by the IB analysis (above), the cell numbers of each group were counted and quantified (below). **h** Hs 819.T cells stably expressing control shRNA, shYAP1#3, or shYAP1#4 were established, then the protein levels of YAP1 and the number of colonies upon YAP1 depletion were determined by IB and colony formation assay, respectively. Data are presented as the mean ± SD of at least three independent experiments in panel (**e, g, h**). One-way ANOVA followed by Dunnett's test was applied for (**e, h**). Two-way ANOVA followed by Tukey's test for (**g**). n.s.: Nonsignificant, * *P* < 0.05, **** *P* < 0.0001.


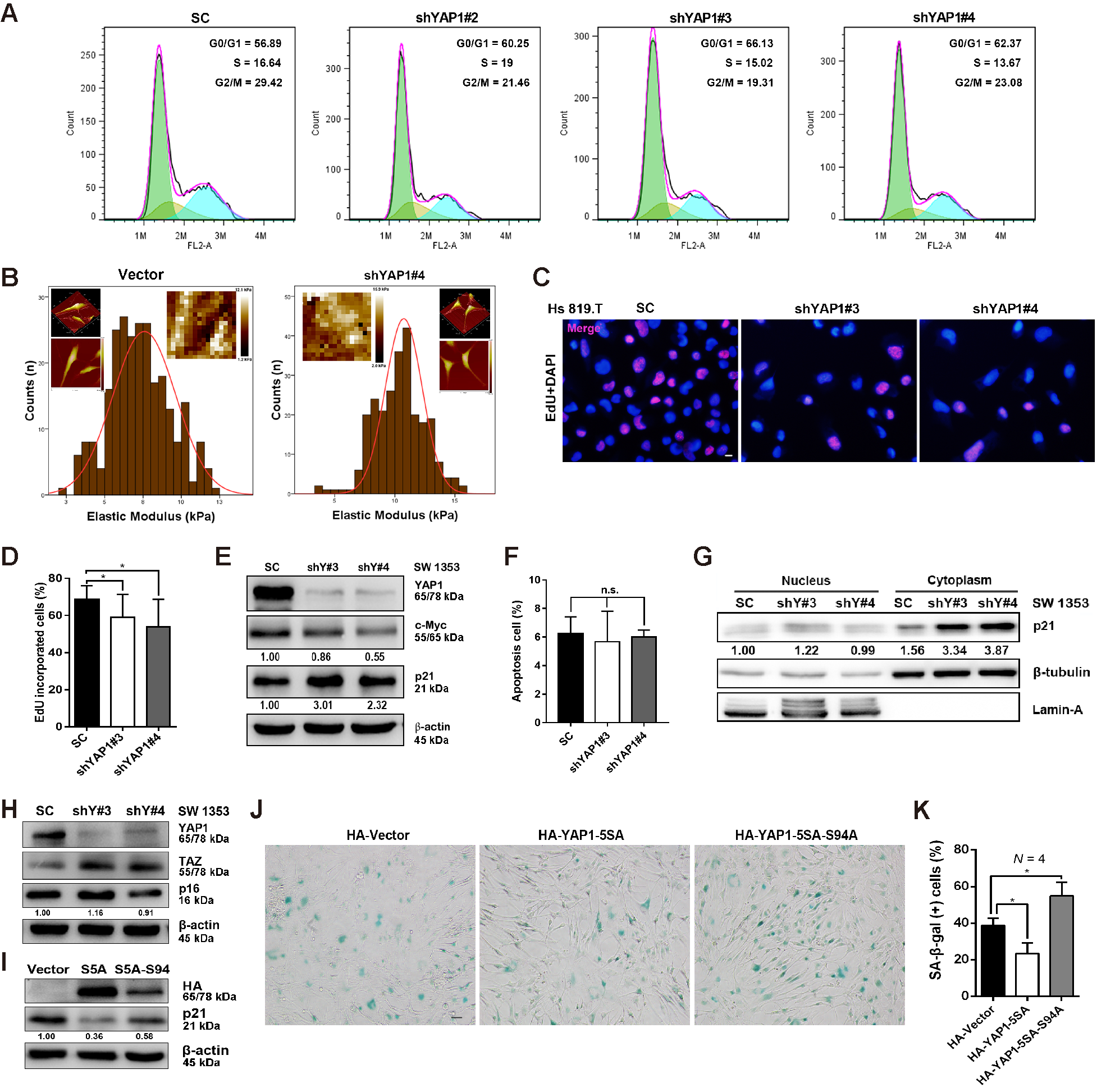


**Fig. S2. YAP1 plays a crucial role in regulating cellular senescence.** **a** Cell cycle distributions of stable control shRNA and YAP1-specific shRNA (shYAP#2, #3, #4) cells were analyzed by flow cytometry. **b** The elastic modulus of stable control shRNA and shYAP#4 cells was analyzed by AFM. **c** Representative images of EdU-incorporated Hs 819.T control shRNA, shYAP1#3, or shYAP#4 cells are shown. DAPI was used as a nuclear counterstain. Scale bars: 10 μm. **d** The percentage of cells in **c** that incorporated EdU was quantified. **e** IB analysis of the indicated proteins, including YAP1, c-Myc, and p21 upon YAP1 depletion in SW 1353 cells. **f** Flow cytometry analysis of apoptotic cells upon YAP1 depletion. **g** SW 1353 cells stably expressing control shRNA or YAP1-specific shRNAs (shYAP#2, #3, #4) were seeded at different cell densities, and IB was applied to determine expression of the indicated proteins. **h** IB analysis of p16 senescence signals. **i-k** Primary chondrocytes were stably transfected with HA-vector (Vector), HA-YAP1-5SA (5SA) or HA-YAP1-5SA/S94A (5SA/S94), and then expression of p21 (i) and the percentage of senescent cells were analyzed (k). Representative images are shown (j). Scale bars: 20 μm. Data are presented as the mean ± SD of at least three independent experiments in panel (**d, f, k**). One-way ANOVA followed by Dunnett's test was applied for (**d, f, k**). n.s.: Nonsignificant, * *P* < 0.05.


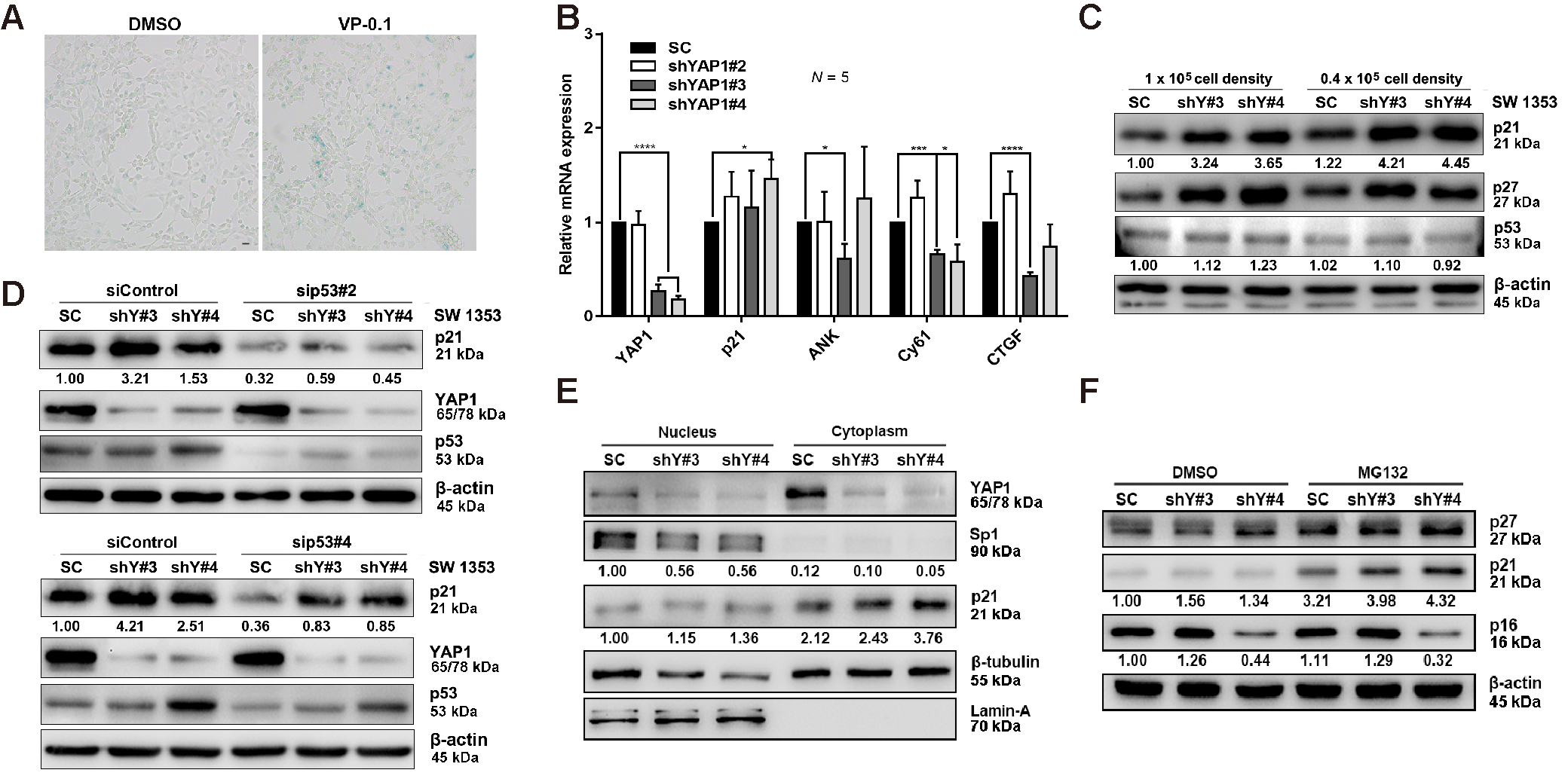


**Fig. S3 YAP1 regulates p21 expression in a p53- and Sp1-independent manner.** (a) SW 133 cells were treated with either DMSO or VP (0,1 uM) and stained with SA-β-gal. (b) The negative regulation of p21 by YAP1 does not occur mainly at the transcriptional level. The mRNA levels of YAP1, p21, ANK, Cyr61, and CTGF were determined by qPCR in the indicated cells. (c, d) The negative regulation of p21 by YAP1 is not p53 dependent. (c) Protein expression of p21, p27, and p53 was detected in the indicated cells at different cell densities. (d) Control shRNA or YAP1-depleted SW 1353 cells were transiently transfected with p53-specific siRNAs (siControl, sip53#2 or sip53#4) for 72 h, and YAP1, p21, and p53 expression was determined by IB. (e) The negative regulation of p21 by YAP1 is not Sp1 dependent. The subcellular localization of YAP, Sp1, and p21 in the indicated cells was determined by IB. (f) Negative regulation of p21 by YAP1 does not occur through the proteasome pathway. Control shRNA or YAP1-depleted SW 1353 cells were treated with DMSO or MG132 for 6 h, and p27, p21, and p16 expression was determined by IB. Data are presented as the mean ± SD of at least three independent experiments in panel (**b**). One-way ANOVA followed by Dunnett's test was applied for (**b**). * *P* < 0.05, *** *P* < 0.001, **** *P* < 0.0001.


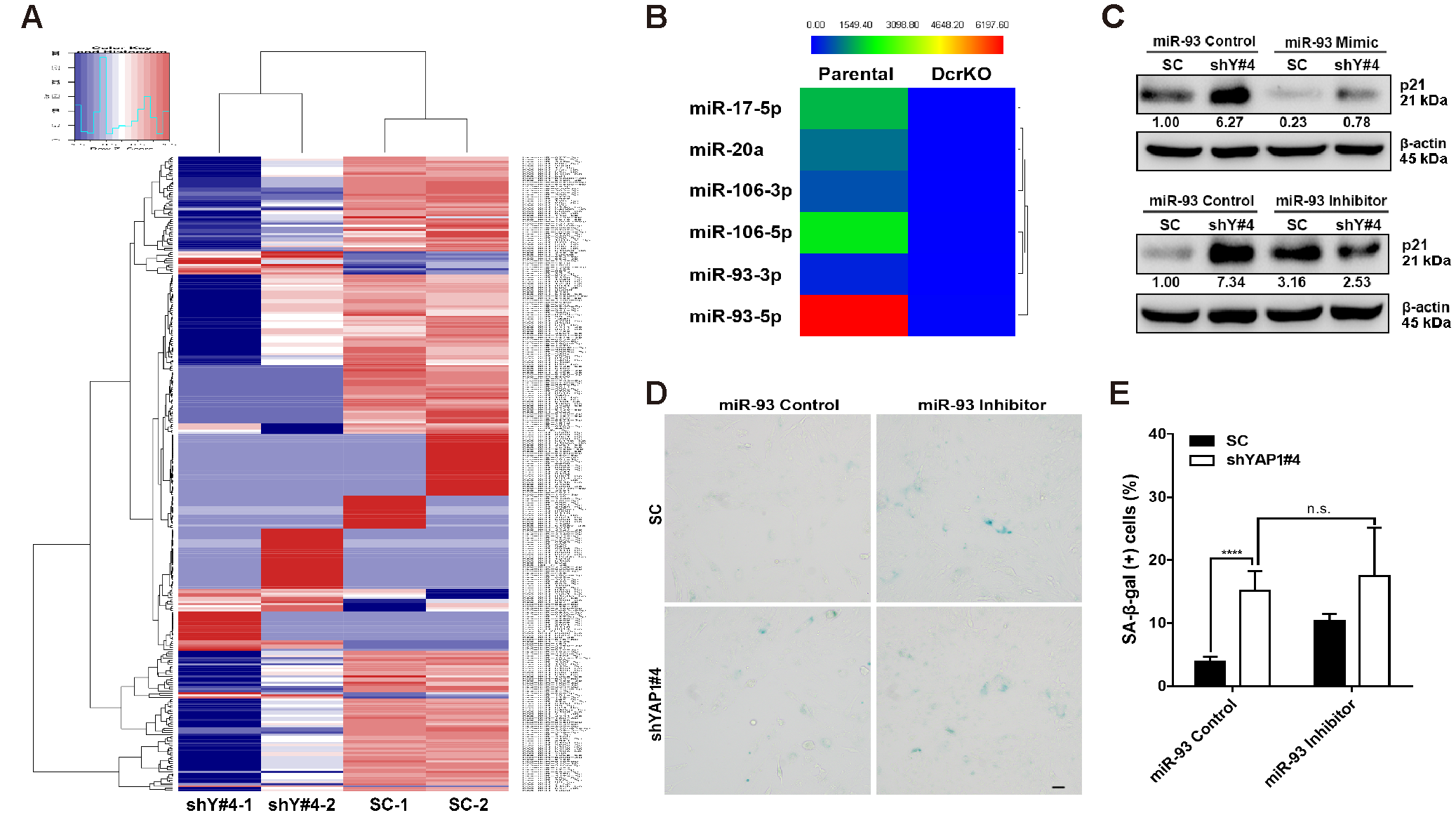


**Fig. S4 The depletion of YAP1 regulates miRNA expression and induces cell senescence.** (a) Duplicated miRNA profiling in control shRNA or shYAP1-depleted (shY#4) cells using small RNA sequencing. (b) Knockdown of Dicer expression caused robust suppression of miR-17 family members (referenced from [53], copyright 2016 Young-Kook Kim). (c) Control shRNA or YAP1-depleted SW 1353 cells were transfected with miR-93 Control, miR-93 Inhibitor or miR93-Mimic, and then the expression of p21 was determined by IB. (d, E) Control shRNA or YAP1-depleted SW 1353 cells were transfected with either miR-93 Control or miR-93 Inhibitor, and representative photos of the senescent cells are shown in (d); scale bars: 20 μm. The percentage of SA-β-gal-positive cells was quantified (e). Data are presented as the mean ± SD of at least three independent experiments in panel (**e**). Two-way ANOVA followed by Tukey's test for (**e**). n.s.: Nonsignificant, **** *P* < 0.0001.


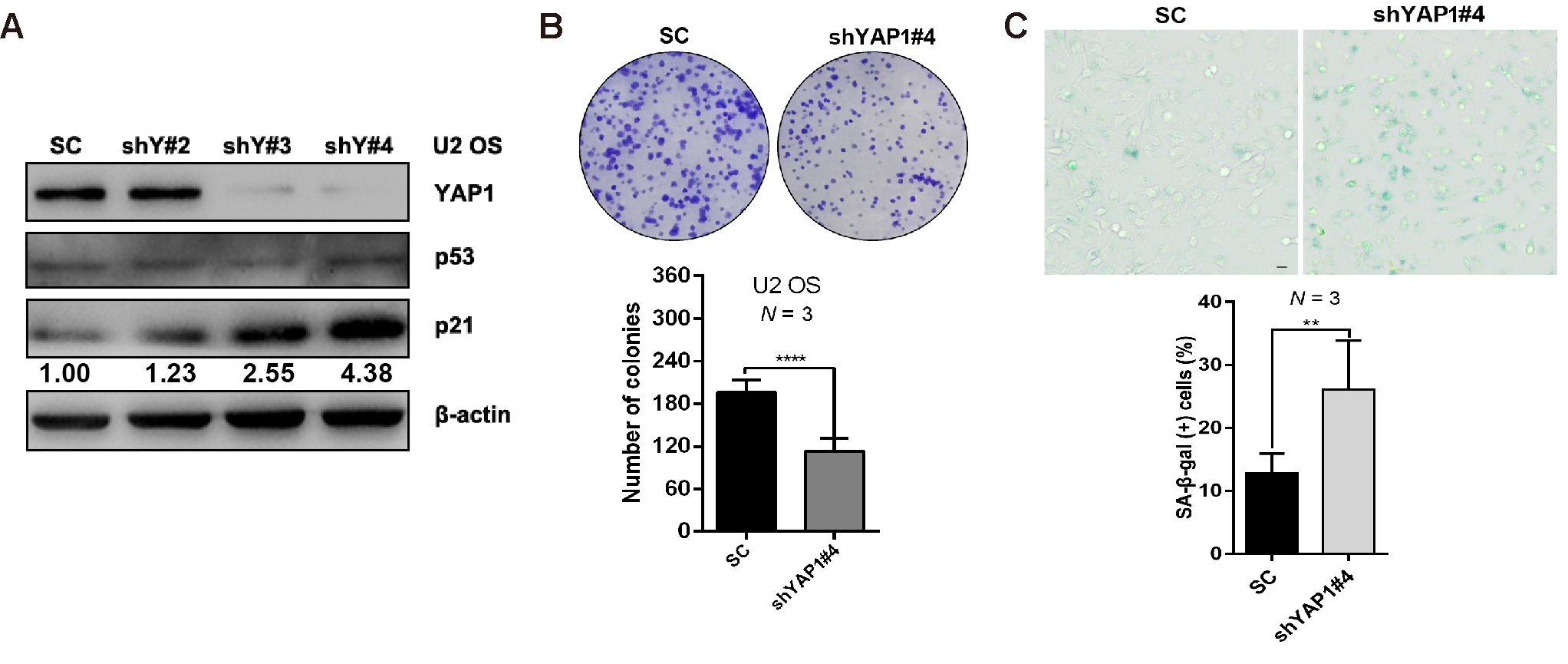


**Fig. S5 YAP1 regulates the cell growth and cellular senescence in osteosarcoma U2 OS cells. a** U2 OS cells were transfected with SC or YAP1-specific shRNAs (shY#2, #3, #4), the expressions of YAP1, p53 and p21 were detected by IB. **b** The representative colony formation images of the SC or YAP1 depletion U2 OS cells (above). The quantification is shown below. **c** The representative SA-β-gal staining positive cells in SC or shYAP1#4 U2 OS cells, The quantification is shown below. Data are presented as the mean ± SD of at least three independent experiments in panel (**b, c**). Student’s *t*-test for (**b, c**). ** *P* < 0.01, **** *P* < 0.0001.
